# Gene Set Enrichment Analysis Reveals Individual Variability in Host Responses in Tuberculosis Patients

**DOI:** 10.3389/fimmu.2021.694680

**Published:** 2021-08-04

**Authors:** Teresa Domaszewska, Joanna Zyla, Raik Otto, Stefan H. E. Kaufmann, January Weiner

**Affiliations:** ^1^Department of Immunology, Max Planck Institute for Infection Biology, Berlin, Germany; ^2^Department for Infectious Disease Epidemiology, Robert Koch Institute, Berlin, Germany; ^3^Department of Data Science and Engineering, Silesian University of Technology, Gliwice, Poland; ^4^Knowledge Management in Bioinformatics, Institute for Computer Science, Humboldt-Universität zu Berlin, Berlin, Germany; ^5^Max Planck Institute for Biophysical Chemistry, Emeritus Group Systems Immunology, Göttingen, Germany; ^6^Hagler Institute for Advanced Study, Texas A&M University, College Station, TX, United States

**Keywords:** tuberculosis, endotypes, individual variability in host response, interferon, immune response, gene set enrichment analysis

## Abstract

Group-aggregated responses to tuberculosis (TB) have been well characterized on a molecular level. However, human beings differ and individual responses to infection vary. We have combined a novel approach to individual gene set analysis (GSA) with the clustering of transcriptomic profiles of TB patients from seven datasets in order to identify individual molecular endotypes of transcriptomic responses to TB. We found that TB patients differ with respect to the intensity of their hallmark interferon (IFN) responses, but they also show variability in their complement system, metabolic responses and multiple other pathways. This variability cannot be sufficiently explained with covariates such as gender or age, and the molecular endotypes are found across studies and populations. Using datasets from a *Cynomolgus* macaque model of TB, we revealed that transcriptional signatures of different molecular TB endotypes did not depend on TB progression post-infection. Moreover, we provide evidence that patients with molecular endotypes characterized by high levels of IFN responses (IFN-rich), suffered from more severe lung pathology than those with lower levels of IFN responses (IFN-low). Harnessing machine learning (ML) models, we derived gene signatures classifying IFN-rich and IFN-low TB endotypes and revealed that the IFN-low signature allowed slightly more reliable overall classification of TB patients from non-TB patients than the IFN-rich one. Using the paradigm of molecular endotypes and the ML-based predictions allows more precisely tailored treatment regimens, predicting treatment-outcome with higher accuracy and therefore bridging the gap between conventional treatment and precision medicine.

## Introduction

Tuberculosis (TB) remains a major threat to human health with 10 million new cases and 1.4 million deaths in 2019 (1). Only a small proportion of the estimated 1.7 billion individuals infected with *Mycobacterium tuberculosis* (Mtb) fall sick with active TB (1). The vast majority of infected individuals contain Mtb in a dormant status resulting in latent TB infection (LTBI), making it difficult to identify the individuals who require treatment (2).

The disparity between progression to TB and continued LTBI constitutes the most obvious kind of variability among Mtb infected individuals. This individual variability exists also on more subtle levels as revealed by gene expression analyses (3–7). Moreover, prospective cohort studies harnessed transcriptomic signatures to predict the risk of TB progression (8, 9). Multiple signatures of TB have been proposed and cross-validated on independent datasets leading to the identification of common motives that were identified in TB patients by most studies with respect to the interferon (IFN) response (3, 4, 7, 10, 11). Patterns of TB-related gene expression regulation however, are heterogeneous and vary within and between studies. Gene-expression trends observed in the majority of TB patients were frequently contradicted by individual TB patients, independently of the applied technology (3). This raises the question whether active TB induces a unique host response pattern or alternatively whether there are multiple, individual host-dependent patterns and whether these are distinct or overlap.

In 2010 Berry et al. proposed a 393- transcript signature of TB which was dominated by IFN-signaling genes (3). The authors investigated transcriptional profiles of TB patients and healthy individuals, and observed that some of the profiles of healthy individuals with LTBI clustered with those of TB patients. Reciprocally, a subgroup of TB patients presented transcriptional profiles that clustered with healthy LTBI and were thus misclassified by their transcriptional signature.

Comparison of the transcriptomes of TB patients to those of healthy individuals gave rise to assumptions regarding the immune response of TB patients, in particular the stronger IFN response as compared to healthy controls. In clinical practice, the most widely used test for Mtb infection is the Interferon Gamma Release Assay (IGRA) which determines the release of IFN γ *in vitro* after stimulation of whole blood (WB) samples with Mtb-specific antigens. The false negative rate of IGRA among Mtb infected individuals is in the order of 15% (12). Thus, blood cells of 15% of the patients do not produce detectable IFN-*γ* levels in response to antigen-specific stimulation. The majority of cohort studies report differentially expressed IFN signaling pathways in TB patients *versus* healthy individuals. Yet, the multiple published ‘TB *vs* healthy’ and ‘TB *vs* LTBI’ signatures only show a limited abundance of shared transcripts. Arguably, this can be explained by different assumptions: (1) expression of different genes may be highly correlated and thus, selecting one or another gene does not influence the performance of the model; (2) different molecular mechanisms dominate the response to TB in different cohorts; (3) various cohorts contain varying numbers of individuals with a certain type of dominant response which influences outcome of comparison of ‘all TB patients’ to ‘all healthy subjects’. For example, the study of Maertzdorf et al. identified JAK-STAT signaling and TLR signaling pathways next to IFN response as dominant in TB (7). In contrast, a study by Verhagen et al. suggested the importance of calcium signaling pathway in TB (13). Studies by Cliff et al. and Cai et al. identified complement system signaling as important correlates of TB (14, 15). It is tempting to speculate that these studies detected different modi in the response to TB: while some cohorts presented dominant regulation of IFN signaling in response to Mtb infection, other presented stronger regulation of calcium or complement signaling. However, a cohort-level analysis cannot determine whether the cohorts comprise patients with cohort-specific responses to TB or alternatively different proportions of patients with specific responses.

We postulated various patterns of host responses to TB, and reasoned that the published WB transcriptomic studies of TB patients are averaged representations of multiple different responses to TB. To test this hypothesis, it is necessary to analyze individual transcriptional profiles between and within independent studies. To address data heterogeneity, we conducted a Gene Set Analysis (GSA) on the integrated transcriptome data with various gene set collections, including pre-defined blood transcriptional modules (BTMs) (16, 17) to reliably identify variability on the level of individual patients. We observed various patterns of gene set enrichment in individuals within single studies, which were reproduced on the level of the meta dataset (MDS) and identified expression patterns within individuals that were significantly correlated with the severity of pathology in the patients’ lungs and characterized by strong enrichment in IFN-response-related modules. Using Random Forest (RF) machine learning (ML) we revealed gene signatures which distinguished between TB patients with different transcriptional response patterns. We then detected additional immune responses, including complement system response, as strongly correlated with the IFN response and therefore contributing to what we defined as “IFN-rich” and “IFN-low” endotypes of TB. To determine whether these two endotypes were a function of time post infection (p.i.), we analyzed data from *Cynomolgus* macaques (18) and observed that even though the IFN response peaked between 20 and 42 days p.i., in various animals the onset of IFN response started at various time points p.i. and lasted for variable periods of time. We further investigated whether additional elements of the host response to TB presenting variable activation in the individual patients can be detected independently of the IFN response. We identified such patterns in the enrichment of metabolic pathways of D-arginine and D-ornithine, as well as in the modules related to insulin and calcium metabolism, using KEGG (19) and MSigDB (20) based GSA followed by principal component analysis (PCA) and eigenvector analysis. Based on these findings, we hypothesize that progression to and severity of TB depend on the variability between individual host responses. Not only does the susceptibility to active disease but also kinetics of the crosstalk between Mtb and the human host differ. Hence, subgroups of TB patients represent different endotypes who would benefit from a personalized host-directed therapy in adjunct to canonical TB drug treatment (2, 21).

## Methods

The overview of the workflow of the study and the used statistical methods can be found in the **Supplementary Figure 1**.

### Data Acquisition and Preprocessing

All utilized datasets are publicly available in Gene Expression Omnibus (GEO) data repository (22). Study-normalized datasets were acquired from GEO *via* the R-package *GEOquery* (23).

Included studies met the following criteria: (i) they contained WB data from untreated TB patients and healthy controls (including LTBI) each; (ii) they contained at least eight samples from TB patients and healthy controls each; (iii) they were performed using platforms which measured expression of at least 16,000 overlapping genes; (iv) they were performed using platforms with annotations available in *BiomaRt* R package (24, 25). Seven datasets were used to create MDS and two independent datasets were used for validation. Additionally, three datasets from sepsis patients who also present strong IFN responses were acquired to validate the presented method.

Data analysis was performed with R (26). The analysis script including all analytical steps is available on the website: (https://github.com/terkaterka/immune-response-to-TB). Datasets were analyzed with R package *limma* for differential expression analysis (27). Microarray data was quantile normalized within single studies to assure comparability. During pre-processing HGNC and ENSEMBL identifiers were mapped to mRNA-array probe names using biomaRt ‘mapIds’ function (*biomaRt* version 2.24.1 (24, 25). Figures were created with the packages *ggplot2* and *UMAP* (28, 29).

We utilized the processed data provided by the respective studies for the integrative meta-analysis. Each dataset was randomly split into 80%/20% partitions with the 80% being used to train the ML algorithm and the 20% being selected for the test set. Only samples classified by the studies as either healthy, LTBI, affected by other diseases (OD) and samples of untreated TB patients were included. MDS was created out of the training sets from each study using only common genes. Nonparametric standardization based on median and interquartile range (IQR) values (Equation 1) was used to standardize the expression values measured in each study, in order to minimize batch effects and heterogeneity between the experiments.

(1) $$e_{i,j}^{’}=\frac{e_{i,j}-mediane_{.,j}}{IQR_{.,j}}$$

Where:

$e_{i,j}^{’}$ – normalized expression value for gene i,

$e_{i,j}$ – expression measurement of gene i,

*IQR_.,j_* – IQR for expression measurement of gene i across all samples.

All utilized data underwent initial quality controls which comprised outliers and artifact-detection and quality-assurance. We ascertained that case and control cohorts clustered according to TB and IFN status and not by their study of origin. Umap-algorithm (29) derived figures depicting the clustering pattern are found in the **Supplementary Figure 2**. To test whether the transformation caused a bias in the GSA (for example significantly changing the findings), we have compared, for each data set, whether the outcome of the GSA changed after applying the transformation. (**Supplementary Method 1**, **Supplementary Figure 3**).

### GSA for Individual Patients

To perform GSA for individual patients, row-wise z-transformation of gene expression values was applied. For each gene, mean expression and standard deviation of its expression were calculated for healthy individuals from every cohort. Subsequently, the mean gene expression of healthy individuals was subtracted from the expression measurements of every individual present in the MDS and the result was divided by standard deviation of gene expression for healthy individuals. The z-score was calculated based on all samples from healthy individuals. Thus, for each patient and gene, the expression z-score is the number of standard deviations below or above the average for healthy individuals. The larger the absolute value of the z-score, the higher the deviation of the expression of that gene from the average in the healthy population.

GSA with CERNO test (30) was performed for every donor on the list of genes ordered by decreasing absolute z-score using tmodCERNOtest function from the R-package *tmod* (31, 32) and BTMs (16, 17).

### Definition of IFN I and IFN II Modules

Two previously published sets of BTMs were utilized (16, 17). A third custom set was generated, based on the classification of genes as IFN I stimulated genes, as IFN II stimulated genes and as genes activated by both IFN I and IFN II signaling pathways according to Interferome v2.0 database (33). The sets consisted of genes which overlapped between originally defined BTMs and genes from the MDS classified by the Interferome v2.0 database either as IFN I inducible genes (IFN I gene sets), IFN II inducible genes (IFN II gene sets) or IFN I and II inducible genes (IFN I and II gene sets). Two additional modules contained (i) all genes classified as IFN I genes and (ii) all genes classified as IFN II genes. The defined module sets are available on the website: (https://github.com/terkaterka/immune-response-to-TB).

### Identification of IFN+ and IFN- TB Patient Groups

GSA was performed on the list of genes from every individual included in MDS sorted by increasing z-score using the three created module sets. Individuals presenting no significant enrichment in any of the IFN I modules were defined as IFN-low and are represented graphically as ‘IFN I-’. Individuals presenting enrichment in at least one IFN I module were defined as IFN-rich and are further represented graphically as ‘IFN I+’. Similarly, the ‘IFN II-’ and ‘IFN I and II-’ individuals presented no enrichment in the IFN II or IFN I and II module set, respectively. Those presenting respective enrichments were defined as ‘IFN II+ or ‘IFN I+ and II+’. Ultimately, the overlaps between study participants classified as IFN I+, IFN II+, and IFN I+ and II+ were analyzed and their classification was compared to IFN+ and IFN- participant groups based on the original BTMs (16, 17).

### Analysis of the Influence of Clinical Factors on IFN Status

To investigate the influence of discrete features (sex, diabetes, HIV, smoking status) on the IFN status the chi-square test was performed and Cramér’s V effect size was calculated. Moreover, the odds ratio (OR) was calculated with 95% CI and the test for OR was conducted (H0: OR=1). For continuous variables (age), the Mann-Whitney test was performed and rank biserial correlation was calculated as effect size.

### Principal Component Analysis

PCA was performed on the MDS as well as on the subset of MDS containing only samples from active TB patients using R-packages *stats*, *pca3d* and *tmod* (26, 31, 34, 35). The fraction of variance (loading) explained by each factorial predictor (TB status, IFN status, study, ethnicity, residence, HIV, OD, mRNA-array technology) was calculated for each principal component (PC; **Supplementary Figure 4**).

### Correlation Between IFN Status and Disease Severity

The dataset GSE19491 (3) was used to compare the IFN status with the disease severity assessed by lung X-Ray studies of TB patients and healthy individuals. The IFN status was determined by transcriptome analysis and a GSA of all participants contained in the study (61 TB patients, 105 healthy individuals including 69 LTBI and 36 non-LTBI, and 274 OD patients). 72 individuals from the study underwent lung X-Ray investigation and were diagnosed as ‘healthy’ (n = 34), ‘minimal disease (n = 14), ‘moderate disease’ (n = 13), or ‘advanced disease’ (n = 11) by physicians blinded to the transcriptome analysis and the clinical diagnosis of the patients (3). The X-Ray based diagnosis was compared with the IFN status assigned on the basis of GSA.

### Machine Learning

#### Random Forest Models With 10-Fold Cross Validation

Random Forest (RF) models were generated using R package *randomForest* (36, 37) to classify TB patients with or without IFN-rich immune response and non-TB individuals including uninfected, LTBI and OD. Class balancing was used to retain the proportion of one case to three control individuals by down-sampling of the majority class. 10-fold cross-validation using R package *caret* was implemented to test the models and their performance was evaluated by creating receiver-operator characteristic (ROC) curves using R package *pROC* (38).

#### Determination of the Signature Size

We ranked the transcripts found in either model according to the amount of statistical importance in the RF model to identify a cut-off for the number of transcripts required to effectively discriminate TB patients from non-TB samples. TB IFN+ and TB IFN- signatures were defined consisting of top (i) 5, (ii) 7, (iii) 10, (iv) 20, (v) 50 or (vi) 200 ranked transcripts, and new models which were trained only on the transcripts that surpassed the cut-off threshold were created. The new models were tested using 10-fold cross validation within the training MDS and their performance was evaluated using ROC plot. The optimal signature size was chosen when the increase of the number of selected transcripts ranked by highest statistical importance did not cause further significant improvement of signature’s area under curve (AUC) in classification of TB patients and non-TB disease controls.

#### Determination of the TB IFN+ and TB IFN- Transcriptional Signatures

For the identification of TB IFN+ and TB IFN- signatures two new class balanced RF models retaining the proportion of one TB to three non-TB cases were trained using the subsets of the complete training MDS subsets containing (i) all TB IFN+ and non-TB (Signature Model 1), (ii) all TB IFN- and non-TB (Signature Model 2). A signature consisting of the 20 top ranking transcripts from the Signature Model 1 was defined as IFN+ signature. A signature of 50 top ranking transcripts from the Signature Model 2 was defined as IFN- signature. Obtained TB IFN+ and TB IFN- signatures were tested on the test MDS and their performance was evaluated by a ROC curve analysis.

#### Validation of the TB IFN+ and TB IFN- Signatures

The obtained TB IFN+ and TB IFN- signatures were tested on the external dataset from Cai et al. (15) and Blankley et al. (39)and their performance was evaluated by a ROC curve analysis. The performances of IFN+ and IFN- TB signatures in detection of sepsis patients were tested to assure that the signatures were disease and not only IFN-response specific.

### Influence of Time Post Infection of *Cynomolgus* Macaques on IFN Status

To determine whether the IFN status in individuals with active TB is the result of time p.i., a longitudinal dataset was procured to assess the changes in the WB gene expression after Mtb infection in *Cynomolgus* macaques (GSE84152 (18);, acquired from the GEO database. The dataset contained mRNA-array data collected from 38 macaques at two time points before Mtb infection and at days 3, 7, 10, 20, 30, 42, 56, 90, 120, 150, 180 p.i., when the diagnosis of TB *vs* LTBI was made. The samples were normalized and z-scores were calculated using the above-described method. GSA using BTMs was performed on samples from individual macaques. The samples were assigned IFN I+/IFN I- status which was compared with their binary clinical diagnosis and severity of lung inflammation.

### Identification of Other TB Endotypes

To determine other endotypes of TB the KEGG (19, 40) and Hallmark (20) gene set collections were investigated by CERNO enrichment method. At first, the results of AUC values from enrichment of each pathway for each patient were extracted. Then, a PCA was performed on a matrix of AUC values and the previously defined IFN+ group was labeled on 2D projection (**Supplementary Figure 5**). To extract other endotypes the eigenvalues of the first PCA component were calculated for all KEGG pathways as well as for all Hallmark MSigDB gene sets and IFN modules across active TB patients. Next, the Spearman rank correlation coefficient was calculated between each KEGG or Hallmark pathway and previously defined IFN modules. Pathways not showing a statistically significant correlation with IFN modules were considered as new TB endotypes. Additionally, we tested whether the proportion of individuals presenting enrichment (adjusted p-value<0.05, Benjamini-Hochberg correction (41); in given enriched gene set and in the IFN gene set was independent using chi square test.

## Results

### GSA Reveals Individual Variability in Transcriptional Profiles Among TB Patients Within and Between Cohorts

**Figure 1:**
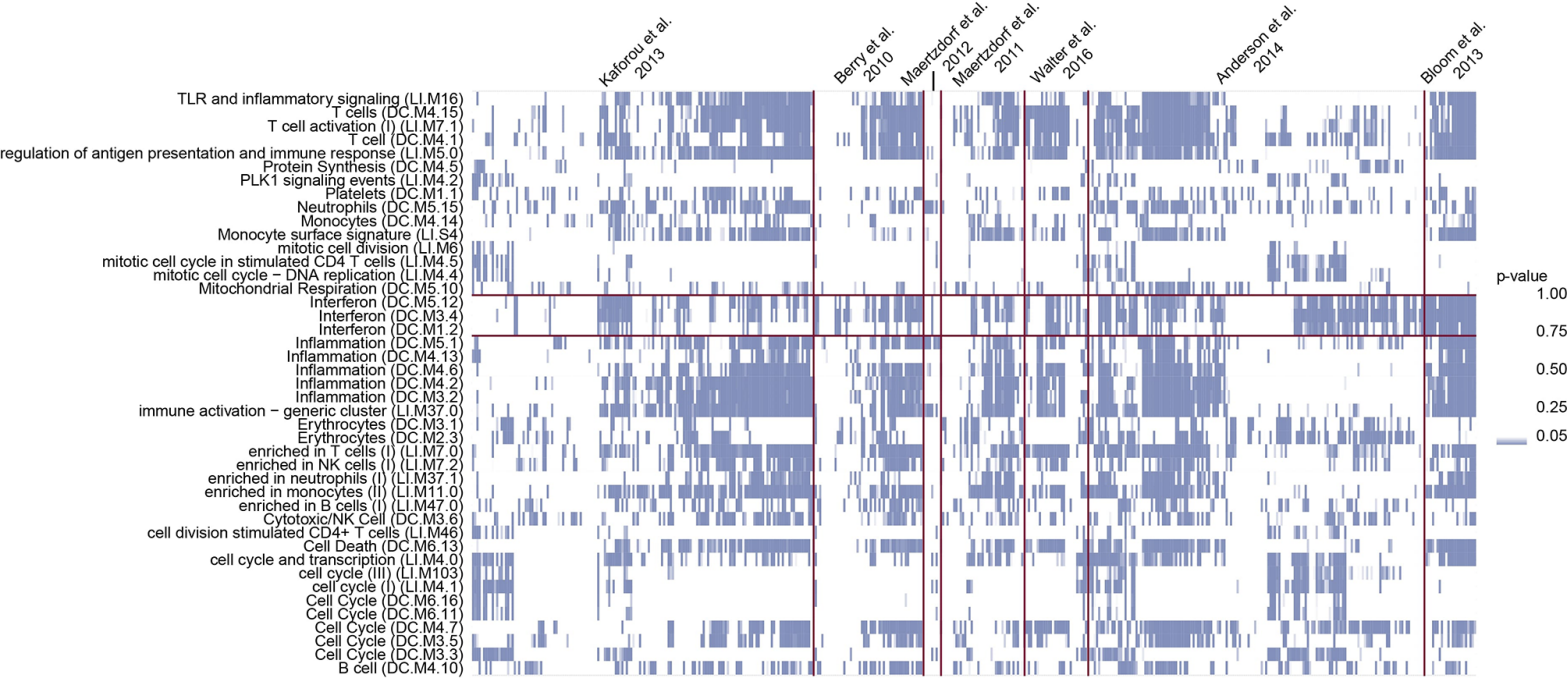
GSA identifies substantial variability among TB patients. Columns show the transcriptional profiles of individual patients and blue squares represent p-values from CERNO test in modules described by the row names. The intensity of the color is proportional to the p-value of enrichment. Study cohorts are separated by vertical purple lines. The horizontal purple lines mark IFN modules. Clearly, in every studied cohort there are individuals presenting enrichment in IFN gene sets as well as presenting no significant enrichment in those sets.

The MDS was generated from seven publicly available datasets using IQR based standardization (Equation 1) for successful integration (**Supplementary Figures 2, 3**). We conducted a GSA for every donor in the MDS on the list of genes sorted by z-score-transformed expression values. Despite visible trends including strong enrichment in T-cell, IFN response and inflammation modules, the enrichment profiles differed between individual TB patients within cohorts which was reproduced between cohorts (**Figure 1**). The group of modules presenting substantial variability between the samples in the enrichment included IFN related modules, generally considered characteristic for TB patients.

### Enrichment of IFN Signaling Gene Sets in the Majority of TB Patients

Given the critical role of IFN type I in the pathogenesis of TB, we focused on the variability of the enrichment of the IFN I-related modules among TB patients since numerous studies have identified IFN I signaling as dominant mechanism in TB (3, 4, 7). The roles of IFN I and IFN II responses in TB differ markedly: IFN I pathways are generally considered detrimental while IFN II is generally assumed beneficial (42). Since many of the IFN stimulated genes (ISGs) can be stimulated by both IFN I and IFN II signaling, we compared the IFN I and IFN II responses in TB patients. We created novel module sets based on the BTMs (16, 17) as well as on the assignment of genes as ‘IFN I inducible’, ‘IFN II inducible’, and ‘IFN I and II inducible’ by Interferome v2.0 database (33). We conducted a GSA with the modules containing genes whose activity was inducible by IFN I signaling and assigned an IFN I status to TB patients based on the following enrichment results: TB patients presenting no significant enrichment in IFN I modules were termed ‘IFN-low’ (‘IFN I-’ for the purpose of graphical representation in this manuscript) and those presenting enrichment were designated as IFN-rich (‘IFN I+’). Out of 457 TB patients, 70% were classified as IFN I+, and 30% as IFN I-. In a similar way we constructed IFN II modules and determined their enrichment in individual TB patients. Intriguingly, enrichment for the modules related to IFN I and IFN II signaling were frequently shared by the same TB patients. Out of 319 TB patients presenting enrichment in IFN I modules (IFN I+ patients), 267 (84%) also showed enrichment in IFN II modules resulting in substantial overlap between IFN I and IFN II induction.

In the following, we use the terms ‘IFN+ patient group’ and ‘IFN- patient group’ based on the enrichment defined using IFN I modules since we focus on IFN I responses, however the high redundancy within the enrichment in IFN II modules implied that that they refer to TB patients with both IFN I and IFN II signaling enrichment in 84% of the cases. Our terminology of ‘IFN+’ status is not to be confused with the abundance of IFN I signaling molecules in blood cells since abundance of ISGs in blood could be related to events at the sites of infection and should be interpreted only as prevalence of IFN inducible transcripts among the significantly regulated genes in TB patients compared to healthy individuals. Similarly, ‘IFN-’ status does not imply a lack of upregulated IFN or ISGs in blood of TB patients but a lack of significant enrichment of IFN modules in an individual sample.

**Figure 2:**
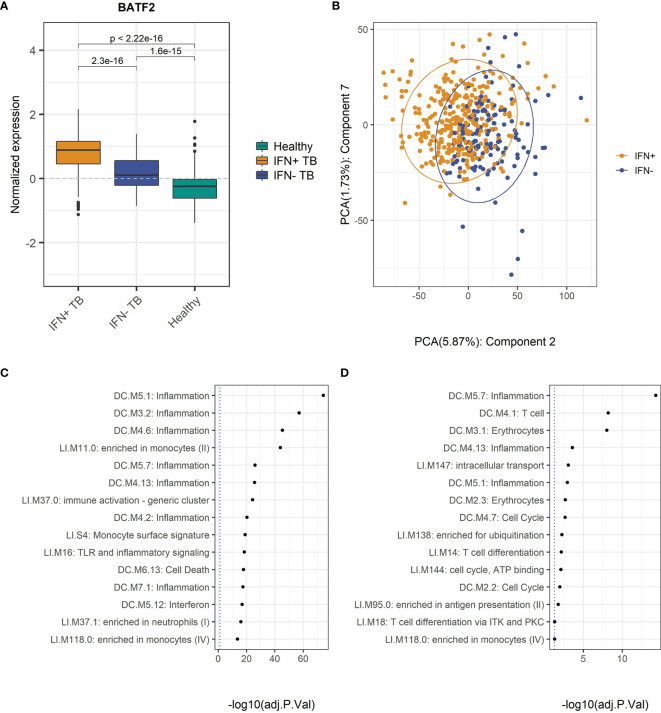
Differences in the gene expression patterns of IFN- and IFN+ TB patients. **(A)** Normalized expression of the BATF2 gene is significantly higher in IFN+ than IFN- TB patient or healthy groups. The p-values were calculated for pairwise comparisons using Wilcoxon test. **(B)** PC2 and PC7 present the difference in the gene expression data of IFN+ and IFN- TB patient groups from the training MDS. **(C)** GSA performed on the list of genes from TB patients from training MDS sorted by decreasing weights in PC2. **(D)** GSA performed on the list of genes from TB patients from MDS sorted by decreasing weights in PC7. GSA indicates that modules related to T cells and inflammation are responsible for differences in IFN status of the TB patients.

To benchmark the modules, we conducted a GSA on the MDS and compared the enrichment status with the differences in the expression levels of several ISGs, among others the BATF2 gene, described as an important ISG (43, 44) which were significantly higher in the IFN+ compared to IFN- patient groups (**Figure 2A**). This indicates that genes identified as crucial for classification of TB patients had different activities in IFN+ and IFN- individuals.

Finally, we tested whether other factors influence predictors of IFN+ status among TB patients. Categorical variables, i.e., sex, diabetes, HIV and smoking status were tested using a chi-square test. Possible association with age was tested using a Mann-Whitney test. For all these investigated factors we did not observe differences and dependencies regarding IFN status (p-value>0.05).

### PCA Supplemented by GSA Indicates Influence of T-Cell and NK-Cell Activity on IFN Status

To further benchmark the implemented division into IFN+ and IFN- groups we tested the differences between the TB patients categorized into the two groups using PCA. The results indicated that although the clusters of IFN+ and IFN- TB patient groups overlapped, the two centers of the clusters were geometrically shifted in regards to each other as best shown by PC2 and PC7 (**Figure 2B**, **Supplementary Figure 6**). GSA applied on genes sorted by their weights in these PCs resulted in a list of significantly enriched BTMs which were dominated by modules related to inflammatory response, induced by IFN type I signaling, and T cells, the main producers of IFN type II (**Figures 2C, D**). This result based on unsupervised analysis strengthens the proposed distinction between gene expression profiles of IFN+ and IFN- TB patient groups.

### IFNR and ISG, but Not IFNα, IFNβ or IFNγ Genes Proper Are More Abundant in the IFN+ Than in IFN- TB Patient Groups

**Figure 3:**
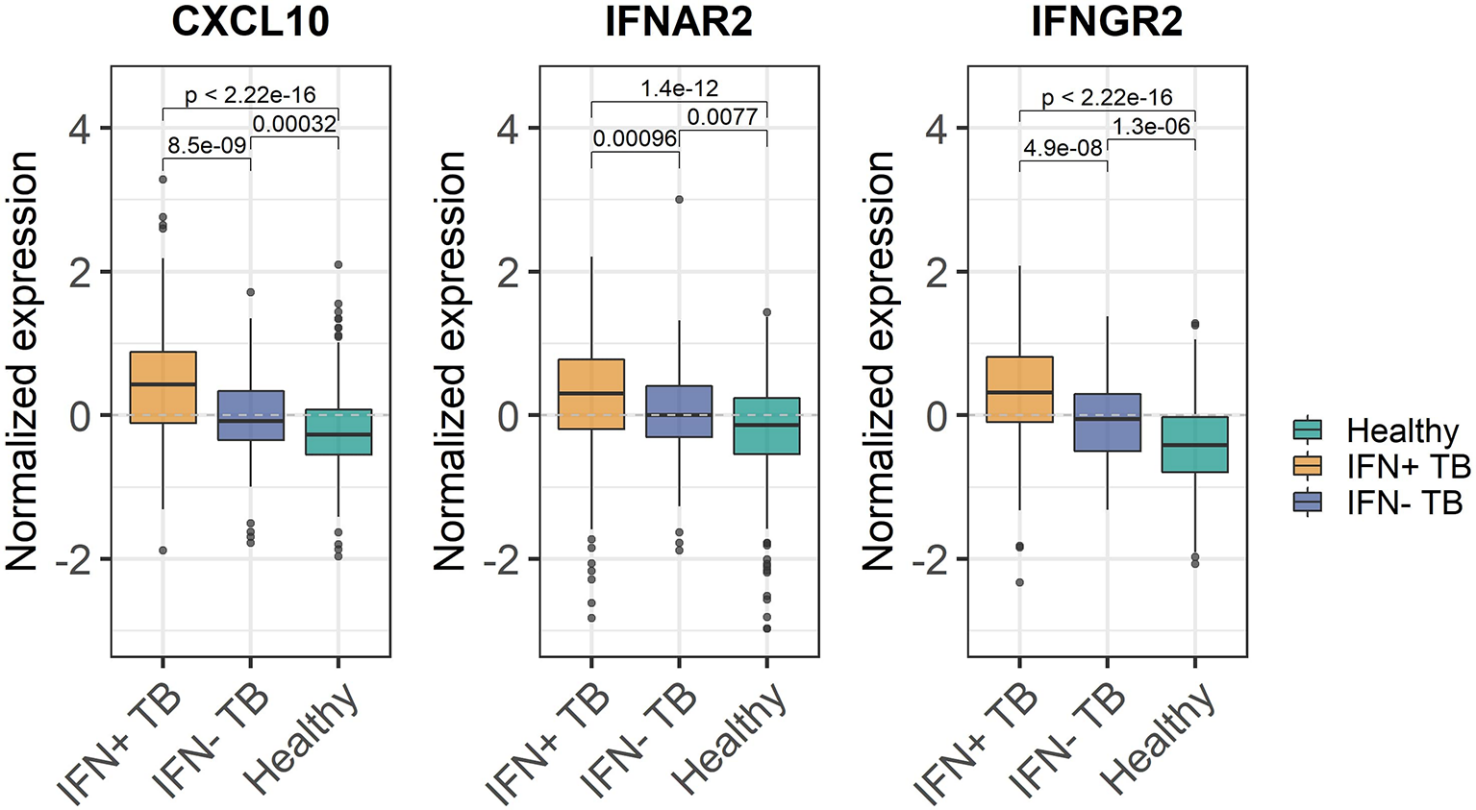
Expression of IFN inducible CXCL10 gene, IFNAR2 and IFNGR2 receptor genes in IFN+ and IFN- TB patient groups and healthy controls. Significant differences have been observed between the expression of IFN-inducible genes between IFN- and IFN+ TB patients. The p-values were calculated for pairwise comparisons using Wilcoxon test.

Enrichment of ISGs in WB does not imply elevated abundance of IFN transcripts in the same tissue but could be related to increased transcription of IFN e.g., at the site of infection. We tested whether the observed enrichment was related to increased expression of the actual IFN α, β or γ genes, IFN receptor genes (IFNR), or ISGs. To avoid using the genes on which the division into IFN+ and IFN- patient groups was based, we identified genes described in IFN signaling pathways, but not included in the original BTMs (16, 17), and determined whether their expression levels varied significantly between IFN+ and IFN- TB patient groups. There were no significant differences of mRNA-transcript levels of the actual IFN α, β or γ genes (IFNA2, IFNB1 or IFNG) between IFN+ and IFN- TB patient groups. In contrast, differences were observed in several of the IFNR (e.g., IFNAR2, IFNGR2) and ISGs (e.g., CXCL10) between IFN+ and IFN- TB patient groups (**Figure 3**, **Supplementary Figures 7A–C**). Hence, differences in IFN+ and IFN- TB patient groups were not a result of increased expression of the actual IFN genes in the WB. Additionally, the differential expression of the IFNR genes and ISGs outside of the defined gene sets confirmed that the transcriptional activation of IFN signaling pathways differed between the patient groups and was not an artifact of the method used for the division.

Interestingly, despite IFN type I and type II signaling pathways being the most studied in the immune response to TB, we found that also IFN λ receptor gene (IFNLR1), but not IFN λ gene (IFNL) itself, which both belong to type III IFN signaling pathway was significantly overexpressed in IFN+ compared to IFN- TB patients or healthy (**Supplementary Figures 7D, E**). IFN λ has been described to have largely overlapping expression and function to IFN type I and to be ubiquitously expressed on epithelial surfaces such as in respiratory tract (45).

### IFN+ Status Correlates With Severe Lung Pathology in TB Patients

**Figure 4:**
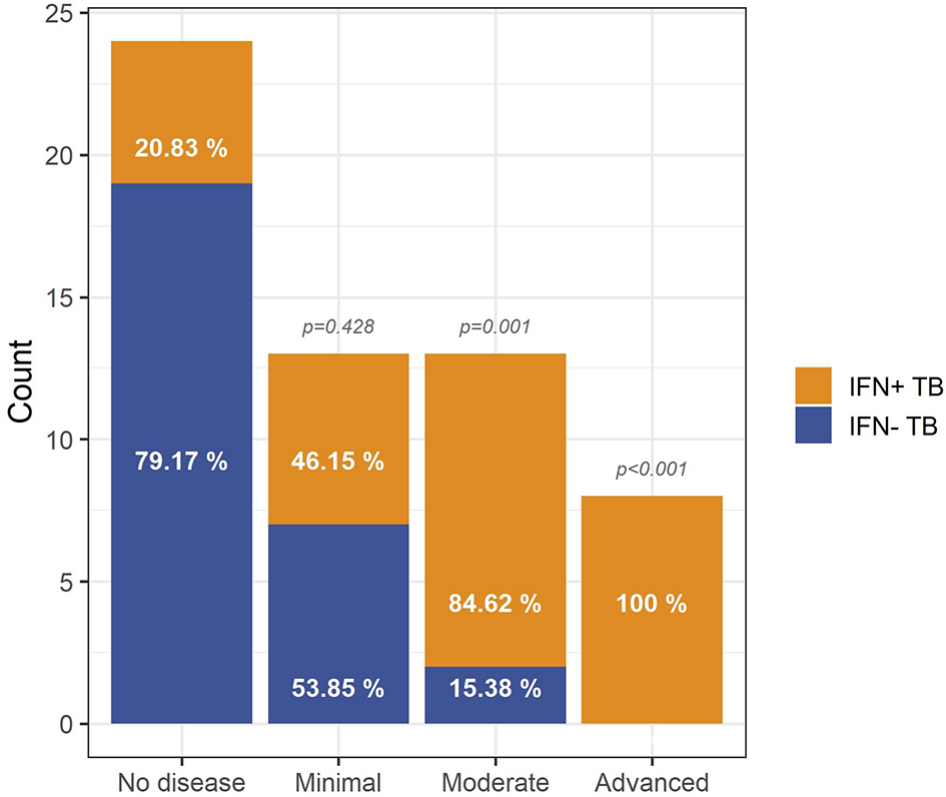
Relationship between IFN status and lung pathology of TB patients. The vast majority of TB patients with moderate (p = 10^-3^) and advanced (p = 4∙10^-4^) pathology is IFN+ whereas absence of pathology is most prevalent in the IFN- TB patient group, indicating that the IFN endotype is associated with a higher level of pathology in TB patients. The p-values were calculated for pairwise comparisons using Fisher’s exact test for count data with Bonferroni correction.

Based on X-Ray images of their lungs, Berry et al. (3) assigned 80 of their study participants to one out of four groups: (i) no pathology, (ii) minimal pathology, (iii) moderate pathology, and (iv) advanced pathology by three independent physicians blinded to mRNA-array data and clinical diagnosis of the donors. We defined the IFN+/IFN- status of these 80 individuals based on GSA and compared them with the X-Ray based on pathologic classifications. 21% of donors classified into ‘no disease’ category presented IFN+ status (**Figure 4**). Among patients with ‘minimal disease’ 46% were IFN+ TB patients, in ‘moderate disease’ category 85% and in ‘advanced disease’ category 100% patients were IFN+. Pairwise comparisons of IFN+ patients in the four categories indicated a correlation between the enrichment in IFN I gene set and the severity of pulmonary pathology of TB patients. This agrees with and extends previous findings of Berry et al. (3) showing that the transcriptional signature of blood cells correlates with extent of pathology in TB patients.

### Gene Signatures in IFN+ and IFN- TB Patient Groups Are Distinct

**Figure 5:**
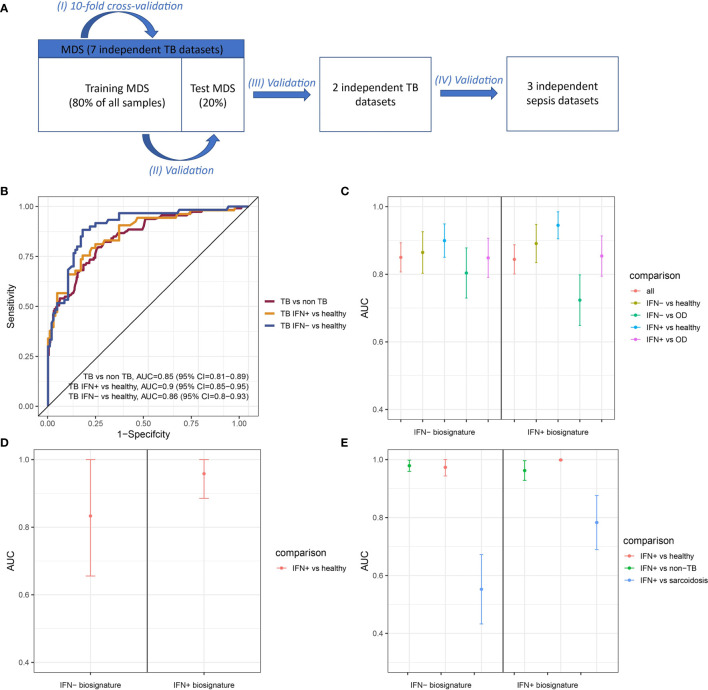
Performance of IFN+ and IFN- signatures on test and validation datasets. **(A)** The validation scheme. To assure the performance of the method four types of validation were used. **(B)** ROC curve presenting the trade-off between sensitivity and specificity of the signature derived from training set consisting of transcriptomic profiles from IFN- TB patients and non-TB controls (healthy, LTBI and OD) and tested on the test set. **(C)** Signatures’ performance in classifying TB patients in the test MDS containing IFN+ TB, IFN- TB, healthy and OD controls. **(D)** Signatures’ performance in classifying TB patients in the validation dataset from China containing only IFN+ TB patient group and healthy controls. **(E)** Signatures’ performance in classifying TB patients in the validation dataset derived from the samples of individuals from London, including IFN+ TB patient group, sarcoidosis patients and healthy individuals. Error bars represent 95% confidence intervals for mean value.

Gene signatures are frequently used to differentiate between TB patients and healthy individuals (8, 9, 46). We created random forests (RF) models trained on subsets of IFN- and IFN+ TB patients and non-TB controls and tested performances of five different sizes of gene signatures derived from these models to determine whether IFN- and IFN+ TB patient groups’ signatures differed. The optimal balance between signature size and model performance was 20 transcripts in case of the IFN+ and 50 transcripts in case of the IFN- signature (**Supplementary Figure 8** and **Supplementary Table 1**). We then derived the two signatures from the training MDS and tested their performances with respect to identification of TB patient groups in the test MDS and two independent TB validation data sets, as well as in the identification of sepsis patients who also present IFN responses. This strategy allowed us to determine whether the signatures were TB-specific and not only IFN-specific. The study scheme is presented in the **Figure 5A** and the performance of the IFN+ and IFN- TB signatures in cross-validation in the **Figure 5B**.

The IFN- signature showed a slightly better overall performance in the identification of TB patients (AUC = 0.86, 95% CI = 0.82-0.90; **Figure 5C**) in the validation set over the IFN+ TB signature (AUC = 0.84, 95% CI = 0.80-0.89; **Figure 5C**). Even though greatest sensitivity and specificity were obtained by the IFN+ signature in the classification of IFN+ TB patients, this model had lower specificity when discerning between IFN- and IFN+ TB patients against OD patients as well as the between the IFN- TB patient groups *versus* healthy individuals.

### Performance of IFN+ and IFN- Signatures on Validation Datasets

We next validated the IFN- and IFN+ RF models on two independent datasets [(15, 47); **Table 1**]. In the first validation set of 21 samples from China containing IFN+ TB patient group and healthy individuals, both models achieved robust discrimination between TB patients and healthy individuals (**Figure 5D**). In contrast, on the set of 202 samples containing a mixed population of IFN+ TB patient group, sarcoidosis patients and healthy individuals of different ethnicities from London, the IFN- TB signature failed to correctly discriminate the IFN- TB patient group from patients with sarcoidosis (**Figure 5E**). Even though the IFN- signature was more universal and stable in discriminating TB patients against healthy and OD controls, it was insufficient for discrimination of TB from sarcoidosis.

**Table 1:** List of publicly available studies acquired for the analysis.

MDS			
Accession number	Citation	Study location	Number of cases
GSE19491	(3)	London, South Africa	54 TB
			96 OD
			93 CTRL
GSE47673	(6)	Malawi, South Africa	215 TB
			194 OD
			175 CTRL
GSE28623	(7)	The Gambia	46 TB
			62 CTRL
GSE34608	(10)	Germany	8 TB
			18 sarcoidosis
			18 CTRL
GSE42834	(4)	London	35 TB
			91 OD
			113 CTRL
GSE39941	(48)	South Africa, Malawi, Kenya	114 TB
			175 OD
			57 CTRL
GSE73408	(49)	USA	35 TB
			39 pneumonia
			35 CTRL
**Validation data sets**			
**Accession number**	**Citation**	**Study location**	**Number of cases**
**TB**			
GSE54992	(15)	China	9 TB
			12 CTRL
GSE83456	(39)	London	45 TB
			47 EPTB
			49 OD
			61 CTRL
**Sepsis**			
GSE13904	(50)	USA	32 sepsis
			67 septic shock
			22 SIRS
			18 CTRL
GSE9960	(51)	Australia	70 sepsis
GSE28750	(52)	Australia	27 sepsis
			30 post-surgical sepsis
			20 CTRL

OD, patients with disease other than TB; CTRL, healthy control patients; EPTB, extrapulmonary TB patients; SIRS, systemic inflammatory response syndrome.

Additionally, we used the TB IFN+ and TB IFN- signatures on datasets from sepsis patients to test whether our signatures did not only detect the IFN responses but also were specific for TB. Both IFN+ and IFN- TB signatures were not sensitive to sepsis (**Supplementary Figure 9**).

### Influence of Time After Infection on IFN Status

We embarked on clarifying whether the IFN status of TB patients was influenced by time p.i. TB patients are diagnosed at various time periods of unknown lengths p.i. Therefore, we interrogated the impact of time p.i. between infection and diagnosis on IFN status. To this end, we harnessed a data set from a controlled Mtb infection experiment of 38 Cynomolgus macaques (18). In this study, animals were infected with Mtb at a fixed time point and blood samples were collected at 11 time points within 6 months p.i. The WB transcriptome had been profiled by mRNA-array at each time point and diagnosis of infection outcome was based on clinical definitions of active TB and LTBI, as well as on the basis of total lung inflammation measured by positron emission tomography - computed tomography (PET-CT) as abundances of [^18^F] fluorodeoxyglucose (FDG) as surrogate marker of severity of pulmonary pathology (18). Sixteen out of the 38 infected macaques developed active TB while 22 remained with LTBI during the time frame of the study (18).

**Figure 6:**
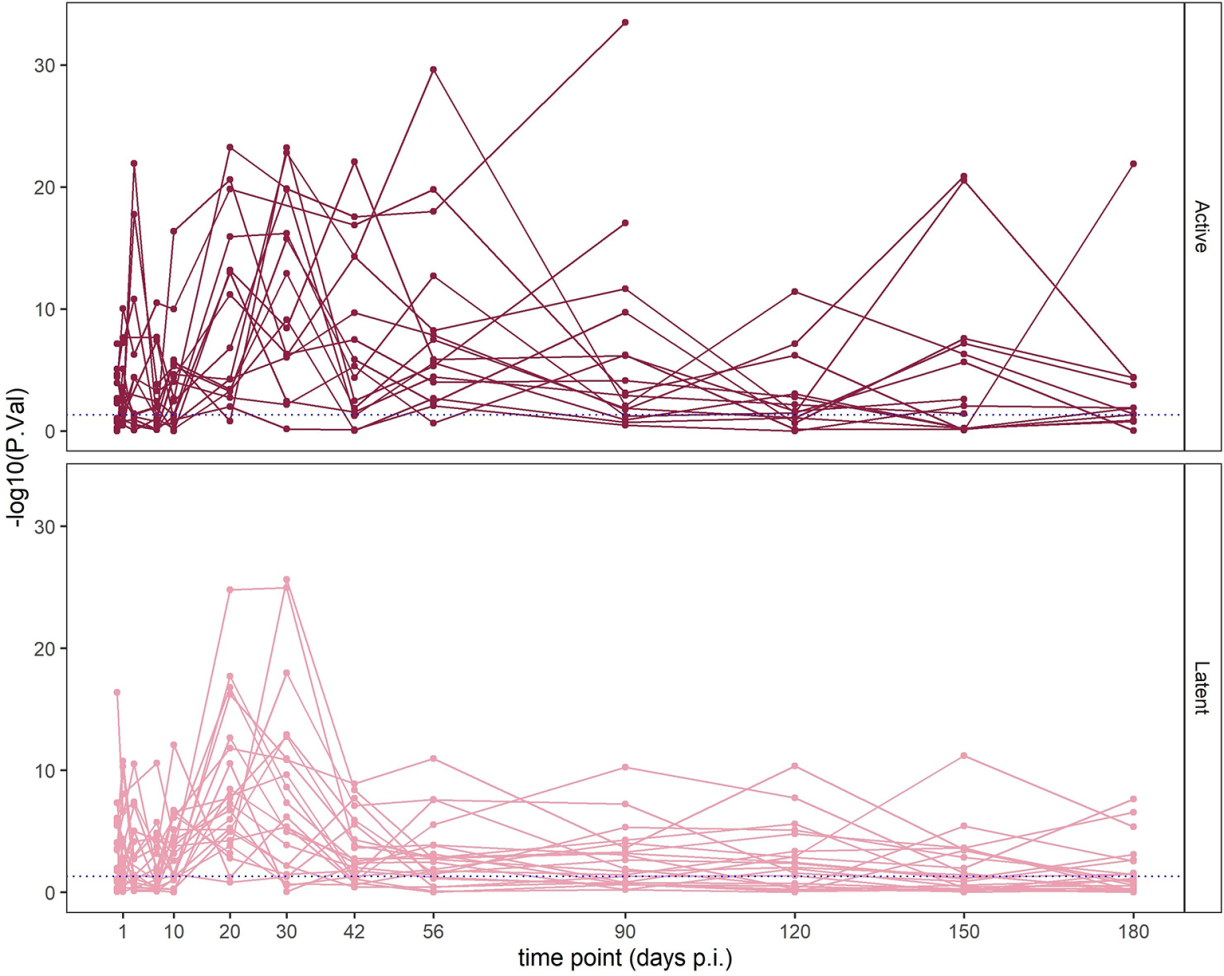
Enrichment of the “IFN I” module in individual macaques over time p.i. Horizontal axis corresponds to time p.i.; vertical axis shows the –log_10_ of FDR in the enrichment (higher values mean lower adjusted p-values; values above the blue line are significant at p < 0.05). Each line corresponds to one animal. Top panel shows macaques which developed active TB disease; lower panel shows macaques which did not develop disease.

At each time point of the study, we assigned these animals either the IFN+ or IFN- status based on the GSA. We observed the peak of the type I IFN response between the 20th and 42nd day p.i. Intriguingly, enrichment in IFN modules was independent from the disease status: between days 20 and 42 p.i., the majority of animals which developed active TB disease as well as those which remained asymptomatic presented strong enrichment in IFN gene sets (**Figure 6**, **Supplementary Figure 10**). Animals progressing to active TB had a prolonged activation of IFN type I response in comparison to animals with LTBI: 75% of them presented enrichment with p-value < 0.0001 at day 56 p.i. compared to 27% with LTBI. Average numbers of time points in which animals with active TB presented enrichment in “IFN type I” gene set with p-value < 0.0001 were 5.9 compared to 4.6 time points in which LTBI animals presented the enrichment in the same module. Additionally, the animals with active TB which had to be at certain time point excluded from the study due to high pathology presented peak of the “IFN type I” gene set enrichment in the last measurement before the exclusion from the experiment, which indicates that the strong IFN enrichment corresponded with heavy disease manifestation. We conclude that the strong regulation of IFN signaling genes is not specific for active TB, yet, it was present for a longer time in animals with active TB. The enrichment in IFN modules was not correlated with time p.i. In fact, in the LTBI animals the IFN response decreased after a peak between days 20 and 30 p.i. (**Figure 6** and **Supplementary Figure 10**) while in animals with active TB the enrichment remained on a high level after the peak between the days 20 and 42 p.i.

### Gene Set Analysis Suggests Additional Potential Endotypes in TB Patients

**Figure 7:**
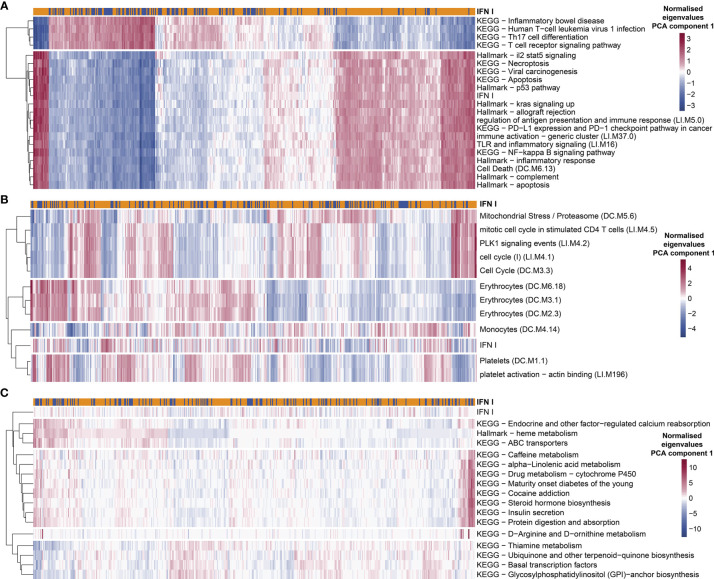
Exploratory analysis suggests additional potential endotypes of TB. The enrichment in KEGG and Hallmark MSigDB gene collections presented variability among individual TB patients which was strongly correlated **(A)**, completely uncorrelated **(C)** or moderately correlated but occurring only in a not-significant proportion of patients with IFN+ status **(B)**. Red color in each plot represents high eigen value from PC1 for particular gene set and patient, while blue shows low eigen value. Colors are normalized row-wise within panels.

Given the differences between groups of TB patients classified as IFN+ and IFN- using GSA we hypothesized that the IFN+ TB endotype can be characterized by additional properties other than intensities of IFN responses, and that potentially also other endotypes of TB patients may exist, which are not correlated with the IFN responses. To this end, we performed an explorative analysis targeted at discovering gene sets which differ in their enrichment between individual patients, accounting for their correlation to IFN responses. We calculated the correlation between eigengenes of the KEGG and MSigDB Hallmark gene set collections and IFN gene set (first PCA components). These databases have a broader (although less specific) scope than the BTMs. Next, we performed GSA using those gene set collections and tested the independence from IFN gene set enrichment in individual proportions. For several of them, the enrichment in individual patients strongly correlated (positively or negatively) with the IFN status. This was the case among others for “Hallmark p53 pathway” module (correlation coefficient of the eigengenes r=0.98), “NF-kappa B signaling pathway” (r=0.96), “Hallmark complement” (r=0.96), “T cell receptor signaling pathway” (r=-0.94), “Th17 cell differentiation” (r=-0.94). Likewise, the proportion of patients presenting enrichment in those modules significantly overlapped with the enrichment in the IFN gene set (p-value from chi^2^ test <0.05). This suggests that the “IFN-rich” endotype is distinguished not only by its pronounced IFN response but also by pronounced NF-kappa B signaling and complement system signaling, while the genes belonging to T cell receptor signaling pathway and Th17 cell differentiation are strongly down-regulated in this endotype (**Figure 7A** and **Supplementary Table 2**). This allowed us to additionally characterize the IFN+ TB endotype not only based on its IFN response but also on other characteristic gene expression features presented by the TB patients with a pronounced IFN response.

We also identified several gene sets which were not correlated with IFN responses, but which nonetheless showed a significant variability in individual patients while at the same time showing differences in enrichment between patients and healthy controls. The enrichment in those modules was much weaker (p-value <0.05 before Benjamini-Hochberg adjustment; p-value >0.05 after the adjustment) and occurred far less commonly among TB patients from different cohorts than the enrichment in the IFN modules. To unravel these, for each given gene set, we tested the correlation of enrichments across all TB patients between the given gene set and the IFN response. This yielded 16 gene sets (out of 388 analyzed in total) which showed enrichment (p-value <0.05 before Benjamini-Hochberg adjustment) in at least 10% of TB patients in the MDS and with no enrichment in the remaining TB patients and, at the same time, no significant correlation with the IFN response (p-value ≥ 0.05; **Figure 7C**). The collection of those 16 gene sets could be grouped into 4 TB endotypes within which the enrichment of the gene sets among the TB patients was correlated (**Figure 7C**). Since such defined endotypes were characterized by correlated, weak enrichment in gene sets with unclear biological connection that we could identify, we named them “Weak TB endotype I-IV” (“WTBE I”, “WTBE II”, “WTBE III”, and “WTBE IV”). The WTBE I, WTBE II, and WTBE III each included at least one gene set which is related to mechanisms that have been previously described as important in TB disease and could lead to diverse clinical characteristics of TB among patients: (1) calcium reabsorption, (2) insulin secretion, and (3) amino acid (D-Arginine and D-ornithine)-metabolism (53–55).

We also observed a spectrum of responses presenting strong enrichment in various subgroups of patients, which only partially overlapped with the IFN endotype. To narrow down the results, we focused only on the responses which were present in more than 10% of the TB patients in MDS, where the 10% consisted of TB patients from at least 6 out of 7 investigated cohorts. Furthermore, to exclude gene sets linked strongly to the IFN endotype, we considered only gene sets which were enriched independent of IFN gene set enrichment. Nine modules presented strong enrichment correlated with r<0.5 and chi^2^ p-value > 0.05: “platelet activation - actin binding”, “PLK1 signaling events”, “Mitochondrial Stress/Proteasome”, “Platelets”, “cell cycle”, “Erythrocytes”, “Monocytes”, “mitotic cell cycle in stimulated CD4 T cells”, “Hallmark heme metabolism” (**Figure 7B** and **Supplementary Table 2**). The role of platelets has been previously described as detrimental in primary progressive TB (56).

## Discussion

Using a novel approach to transcriptome analysis, we discovered individual variability among TB patients across seven cohorts, in particular with respect to IFN responses. Principally, IFN responses are induced by IFN type I or IFN type II which cause harmful or beneficial sequelae, respectively, in TB. We found that IFN responses were not equally distributed amongst TB patients. Rather, in a subgroup of TB patients, IFN responses were the dominant immune responses, which we defined as IFN+ group of TB patients while they were less pronounced in the group defined as IFN- group of TB patients. The distinct population of TB patients who did not develop IFN responses detectable by GSA presented less severe lung pathology. The findings were complemented by non-human primate studies which revealed that enrichment in IFN gene sets is not a result of time p.i. Finally, we determined that the gene signature from the IFN- TB patient group provided comparable, slightly higher sensitivity and specificity for overall diagnosis of TB, primarily because of better classification of IFN- TB patients from OD patients, and analyzed further mechanisms differentially regulated between subgroups of TB patients which should be further explored as new potential TB endotypes with different underlying host immune responses. Our results revealed various enrichment patterns among TB patients within cohorts, which could be reproduced between cohorts. The dominant patterns included enrichment of modules including T cells, B cells, innate immunity, IFN signaling, monocytes and erythropoiesis. However, 30% of the TB patients did not present enrichment in IFN related modules.

Two types of IFN signaling are considered crucial for the outcome of TB: (i) the IFN I signaling pathway is thought to be mostly detrimental and (ii) the IFN II pathway is considered to play a major role in protection (42). Yet, our analyses revealed that the majority of TB cases shared IFN I and IFN II response enrichments indicating that detrimental and beneficial mechanisms coexist in active TB disease in a fine-tuned way. Consistently, lung pathology was far more prevalent in the IFN+ than IFN- TB patient groups. Possibly, the balance between both types of IFN determines the outcome of the infection.

Unsupervised analysis identified that samples collected from IFN- and IFN+ TB patient groups cluster together but are shifted with regard to each other. GSE on the weights of genes revealed contribution of T cells, which are potent producers of IFN γ and IFN α cytokines (42). PCA of samples from TB patients showed that even though the data had been normalized, differences between datasets from different studies still stratified the data (**Supplementary Figure 4D**).

Our analysis demonstrates that differences in the enrichment of IFN related modules is not a consequence of varying abundances of IFN α, IFN β or IFN γ in WB since the IFN+ and IFN- patient groups presented similar expression of IFN α, IFN β and IFN γ genes. Rather, significant differences in the expression of IFN α and IFN γ receptors and ISGs such as CXCL10 between the IFN+ and IFN- TB patient groups were critical. We conclude that regulation of gene expression in IFN+ and IFN- TB patient groups was not caused by differential expression of IFN α, β or γ, but by differential expression of IFNR genes and ISGs.

The abundance of the transcript BATF2 contributed to the differences in enrichment observed between IFN+ and IFN- TB patient groups. The BATF2 levels were significantly higher in IFN+ than IFN- TB patient groups. This leucine zipper transcription factor has been shown to exacerbate lung pathology in an experimental mouse model (43) and has been suggested as a single biomarker for TB (44). We conclude that BATF2 is regulated by IFN and primarily detrimental in TB.

We defined diagnostic signatures of IFN+ and IFN- TB patient groups using ML methods. The selected IFN+ signature comprised 20 transcripts, while the optimal IFN- signature consisted of 50 transcripts. 7 transcripts were present in both IFN+ and IFN- TB signatures: GBP5, AIM2, GBP2, POLB, WARS1, LHFPL2, DUSP3. Several of these genes are related to IFN-signaling which emphasizes the important role of IFN in TB even in patients in whom IFN signaling is enriched marginally.

The IFN+ and IFN- signatures were assessed on the test MDS and validated on two independent datasets, one including healthy controls and TB patients from China (15) and one including healthy controls as well as TB and sarcoidosis patients from an ethnically diverse population in London (47). The signature derived from the IFN+ TB patient group was highly sensitive and specific towards IFN+ TB patient groups, however its performance was unsatisfactory with respect to discriminating between the IFN- TB patient group from healthy, and in particular against patients with OD. The IFN- TB signature presented slightly higher AUC values for identification of TB patients in the test set, but lower ones in the two validation sets. To the advantage of the IFN- TB signature, its classification was characterized by similar sensitivity and specificity for discriminating IFN- and IFN+ patient groups against healthy, OD and all non-TB controls. The exception was the discrimination of TB *versus* sarcoidosis patients, which was only satisfactory for the IFN+ TB signature. TB and sarcoidosis have been shown to present a remarkably high overlap between biomarkers that discriminate *versus* healthy controls (10). In summary, our results demonstrate that: (i) the IFN+ and IFN- TB patient groups were characterized by different signatures; (ii) the IFN- TB signature identified the IFN+ TB patient group whereas the IFN+ signature failed to diagnose the IFN- TB patient group; (iii) even though the TB IFN- signature was more stable in detecting TB patients, it failed to differentiate between IFN+ TB patient group and sarcoidosis patients.

Using datasets from the macaque TB model from Gideon et al. (18) we probed whether the IFN status in TB can be explained by the time p.i. The heterogeneity in IFN responses was observed in the animals independent of the severity of TB disease and did not correlate with the time p.i. We observed a slight overall increase of IFN responses between 20- and 40-day p.i. corresponding with the findings of Gideon et al. (18). During this time period adaptive immunity kicks in (57). The effect size and p-value of enrichment in IFN gene sets in given animals did not correspond with the establishment of active TB or LTBI and varied among diseased as well as LTBI animals. Thus, a strong IFN response upon Mtb infection did not correlate with a particular stage of infection and progression to active disease, and its dynamics was highly individual.

In the study of Berry et al. (2010) the transcriptomes of eight out of 52 LTBI patients clustered with profiles of TB patients and four out of 21 TB patients presented transcriptional profiles resembling those of LTBI. Similar observations were reported by Blankley et al. (47) who determined transcriptional profiles of 61 healthy donors, 47 patients with extrapulmonary TB, 45 patients with pulmonary TB and 49 donors with sarcoidosis. They found that a subset of patients with TB clustered with the healthy donors. The GSA enrichment scores of modules related to IFN responses reflected the extent of symptoms presented by the study participants (47). As suggested by the recent studies of Zak et al. (8), Suliman et al. (9) and Singhania et al. (58), classification as ‘TB’ and ‘non-TB’ by clinical assessment and transcriptomic profiling can be confounded by subclinical incipient TB and early stages of progression to active TB disease. Transcriptomic profiling, but not clinical diagnosis, identifies subclinical TB and risk of progression to active TB within 12 months (8, 9). Our study further emphasizes the need to better define the continuum from LTBI to subclinical TB to active TB disease and also to distinguish between different endotypes of active TB.

To this end, we next explored the possibility of further novel, molecular endotypes of TB, which are not directly linked to IFN responses. We found that TB patients differ in the activity of genes associated with calcium signaling, insulin signaling and amino acid metabolism. These findings introduce an exciting new avenue of exploring pathways linked to TB. We are aware that our results are based on lack of an observed correlation with the IFN response – and thus might indicate lack of evidence for association with IFN rather than evidence of lack. However, given the large number of patients on which we based our study, we feel reasonably confident that if such effect exists, its magnitude must be small. Clearly, as these findings are based on an exploratory analysis, a further validation targeted directly at our hypotheses will be essential. The concept of endotypes to describe subgroups of patients on the basis of distinct transcriptomic, epigenetic or metabolic features has been applied to several diseases, most recently also to TB (21). A combination of a specific endotype and certain environmental factors has important impact on the disease phenotype. It is likely that different endotypes require distinct types of host-directed therapy (2). Our data support the definition of molecular endotypes of TB with the IFN subtypes described here as major but not exclusive contributors.

Although our study provides deeper insight into individual variability among TB patients at the level of gene expression, there are limitations: first, human cohorts are highly variable due to numerous factors including genetic variability, conditions of life and circumstances of infections (such as coinfections, the frequency of reinfection with Mtb, and time to and reason of diagnosis). Additional confounders include varying study designs and conduct, as well as technical variation. To partly account for these confounders, we validated our results in several ways including cross-validation and leaving 20% of the acquired studies unprocessed for independent testing, acquisition of independent validation datasets and testing the gene signatures of IFN+ and IFN- TB on a different disease - sepsis. Application of our data collection, normalization and analytical methods in numerous external datasets revealed that the proposed analytical framework is robust and can be used in other multi-cohort studies. Our dataset collection selected out of published TB datasets and newly defined sets of IFN I, IFN II as well as IFN I and II inducible genes can be accessed on the website: (https://github.com/terkaterka/immune-response-to-TB).

An important conclusion from our study is that TB disease signatures are confounded and biased if they do not account for individual variability between study participants. The complexity of human TB presenting a continuum from LTBI to subclinical TB to different forms of active TB disease implies that the assignment of TB patients into one of the two general classes: ‘diseased’ or ‘healthy’ is insufficient. Future focus should lie on detection and exploration of other than IFN determinants of the course of TB in individual patients. It is thus most likely that active TB manifests as different endotypes which may need personalized treatment regimens as alternative or in adjunct to canonical treatment regimens. Notably, host-directed therapy will most likely differ in these two endotypes of active TB described here with the IFN+ group likely benefiting from IFN dampening and the IFN- group likely from IFN promoting therapy.

## Data Availability Statement

Publicly available datasets were analyzed in this study. This data can be found here: All of the used, publicly available datasets are referenced in the manuscript. The datasets are found in the Gene Expression Omnibus (GEO; https://www.ncbi.nlm.nih.gov/geo/) data repository.

## Author Contributions

TD collected the data sets, performed data analysis, and wrote the main manuscript file. JZ was incorporated into calculations and algorithm development that could be described as the performance of tasks in the technical informatics discipline defined by the Polish Ministry of Science and Higher Education. TD and JZ prepared the figures. RO performed Umap analysis of the datasets. JW designed and supervised the study with additional input from SK. All authors contributed to the article and approved the submitted version.

## Funding

This work received intramural funding from the Max Planck Society to SK. This work was partially supported by the Silesian University of Technology grant for Support and Development of Research Potential (JZ).

## Conflict of Interest

The authors declare that the research was conducted in the absence of any commercial or financial relationships that could be construed as a potential conflict of interest.

## Publisher’s Note

All claims expressed in this article are solely those of the authors and do not necessarily represent those of their affiliated organizations, or those of the publisher, the editors and the reviewers. Any product that may be evaluated in this article, or claim that may be made by its manufacturer, is not guaranteed or endorsed by the publisher.
