# Supplementary material for: Gene Set Enrichment Analysis Reveals Individual Variability in Host Responses in Tuberculosis Patients
Source: Front Immunol. 2021 Aug 4;12:694680. doi: 10.3389/fimmu.2021.694680 (PMC8375662; doi:10.3389/fimmu.2021.694680)
Supplement: Supplementary file 1 [file DataSheet_1.docx]

**Supplementary Material**

## Supplementary Method:

## Differential gene expression calculation and gene set analysis

Genes that were significantly differentially expressed between healthy individuals or individuals with other diseases (OD) and individuals suffering from TB (p-value threshold 0.05) were identified using limma lmFit function (Ritchie et al., 2015) with the factors: stimulus type (‘TB’, ‘OD’ and ‘healthy’). The p-values were calculated based on moderated t-statistics and corrected for multiple testing using the Benjamini-Hochberg method (Benjamini & Hochberg, 1995). Differential regulation of genes was computed on the datasets normalized by authors as well as separately for each study after normalization. GSA was performed for every dataset before and after normalization using R package *tmod* (Weiner & Domaszewska, 2016) and the results were compared based on the presence of enriched modules before and after normalization.

## Supplementary Figures:


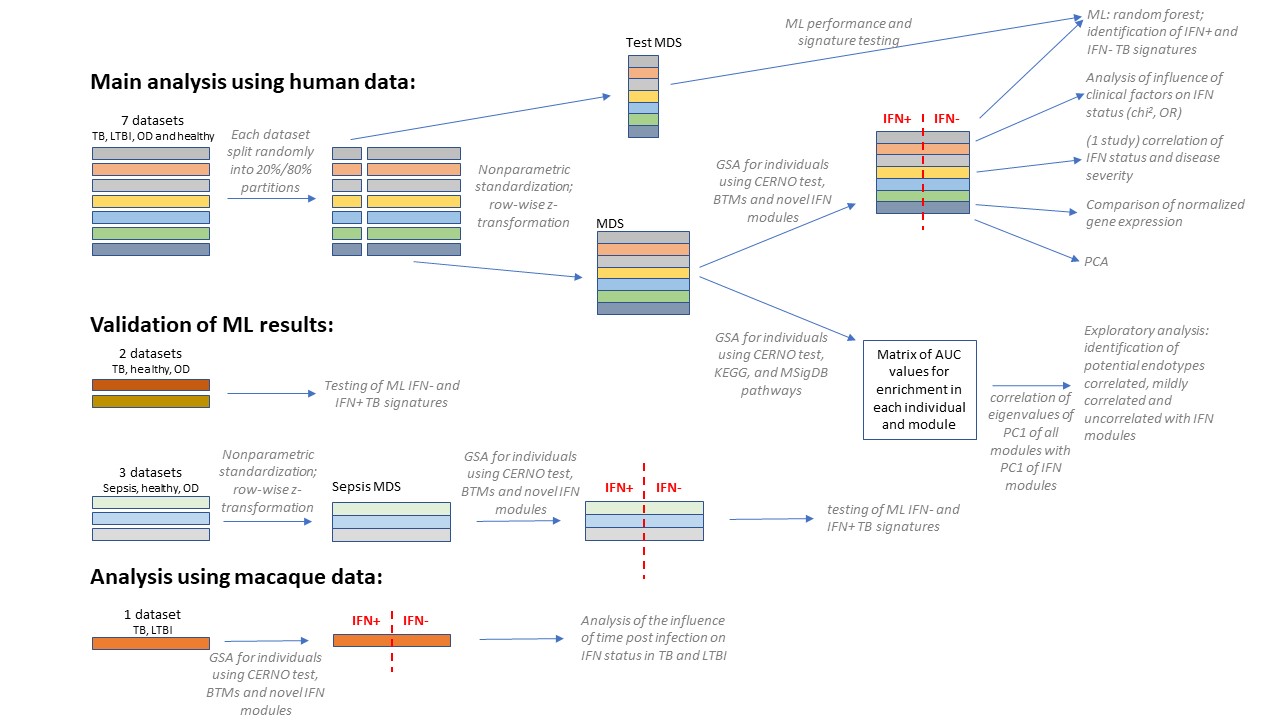


**Supplementary Figure 1: overview of the study and the used statistical methods.** 7 human datasets were integrated in a meta-dataset (MDS) and used for the analysis of individual variability in immune responses of TB patients. Additional two TB and three sepsis datasets were used for the validation of the ML methods. One macaque dataset was used to investigate the influence of time post infection on IFN status.


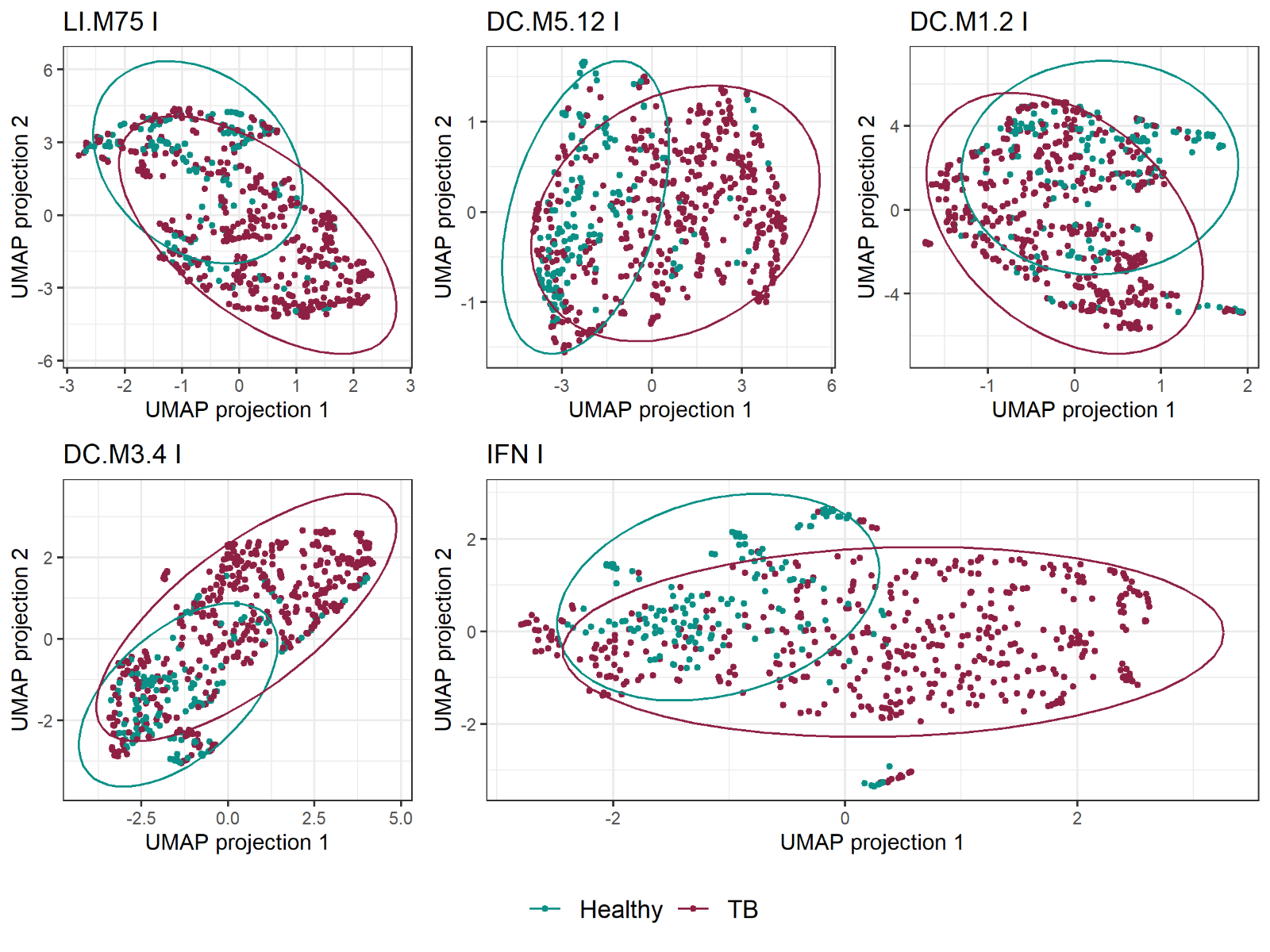


**Supplementary Figure 2:** **UMAP visualization of cohort separation.**The UMAP algorithm stochastically projects pair-wise sample differences within the first PCs which cumulatively explains 90% of variation in dataset and project the higher dimensions onto a two dimensional plain as shown here. Red color represents active TB cases whereas blue represent healthy individuals.  Despite significant noise introduced by the integration of heterogeneous datasets case and control cohorts can be separated by the sum of their variation within higher PCs. Thus, the biologically relevant information was retained and differences between case and control cohorts with respect to the TB status existed for all modules.

 
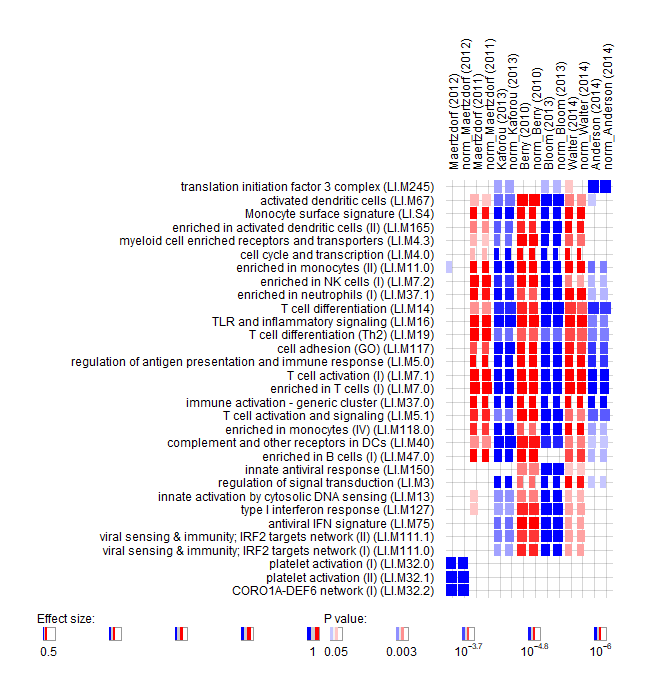


**Supplementary Figure 3: GSA performed on all the studies before and after the normalization**. The names of columns are named after the first author of the study. The column names of the columns presenting enrichment after the median and IQR normalization start with ‘norm_’. The bars present enrichment in the modules described in the row names. The bar size is proportional to effect size and the intensity of the color is proportional to decrease in p-value of the enrichment. The red and blue colors are used to facilitate distinguishing subsequent studies.

 
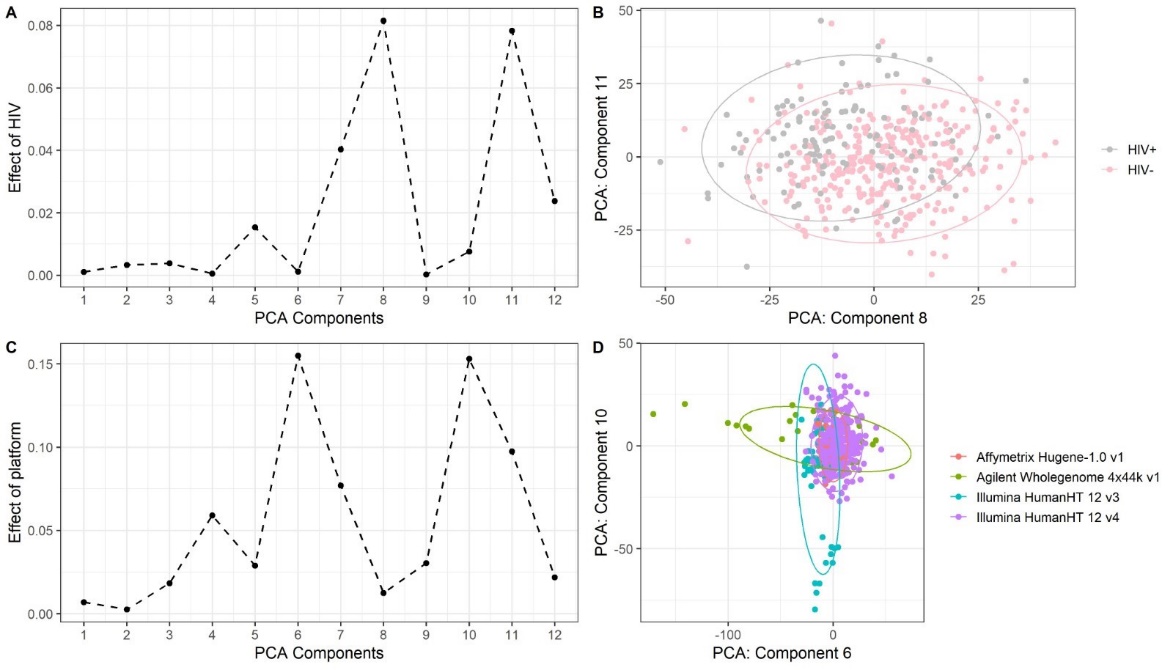


**Supplementary Figure 4: PCA of the matrix of gene expression of TB patients from the training MDS.** 75% confidence interval ellipses for the groups are shown. (A) Fraction of variance explained by HIV status as a predictor for each of the first 12 PCs of the gene expression matrix from TB patients from the MDS calculated using 100-times randomization. PC8 and PC11 explain the biggest fraction of the variance. (B) PC 8 and 11 colored by HIV status. (C) Fraction of variance explained by the used mRNA-array platform as a predictor for each of the first 12 PCs of the gene expression matrix from TB patients from the MDS calculated using 100-times randomization. PC6 and PC10 explain the biggest fraction of the variance. (D) PC 6 and 10 colored by used mRNA-array platform.


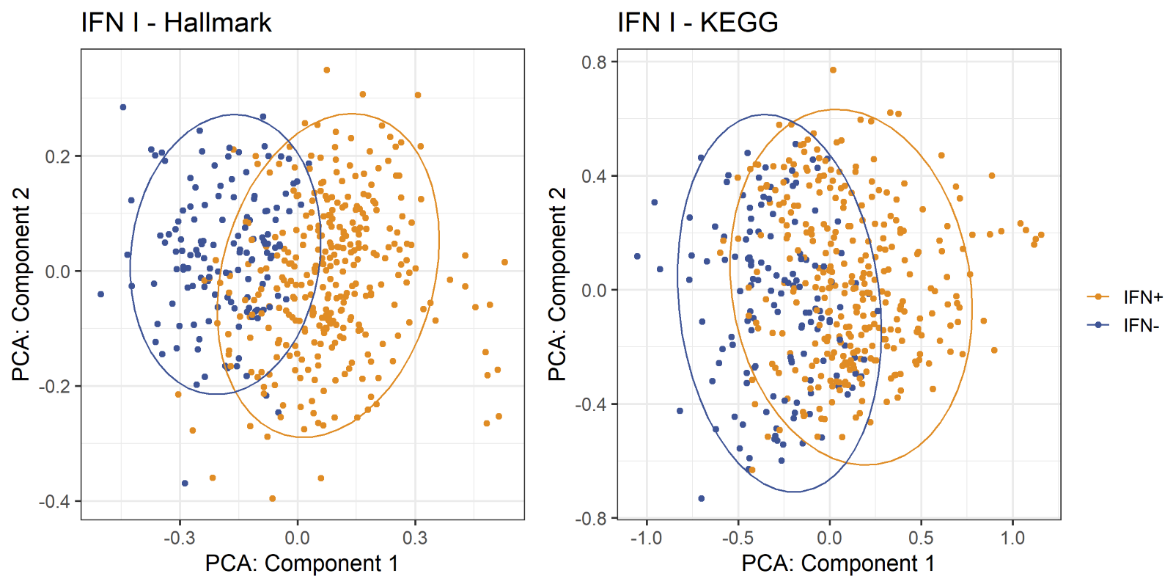


**Supplementary Figure 5: PCA on a matrix of AUC values for the investigated gene sets and all TB patients. PC1 is plotted vs. PC2 and colored by IFN status**. A – MsigDB Hallmark gene sets; B-KEGG gene sets.

  
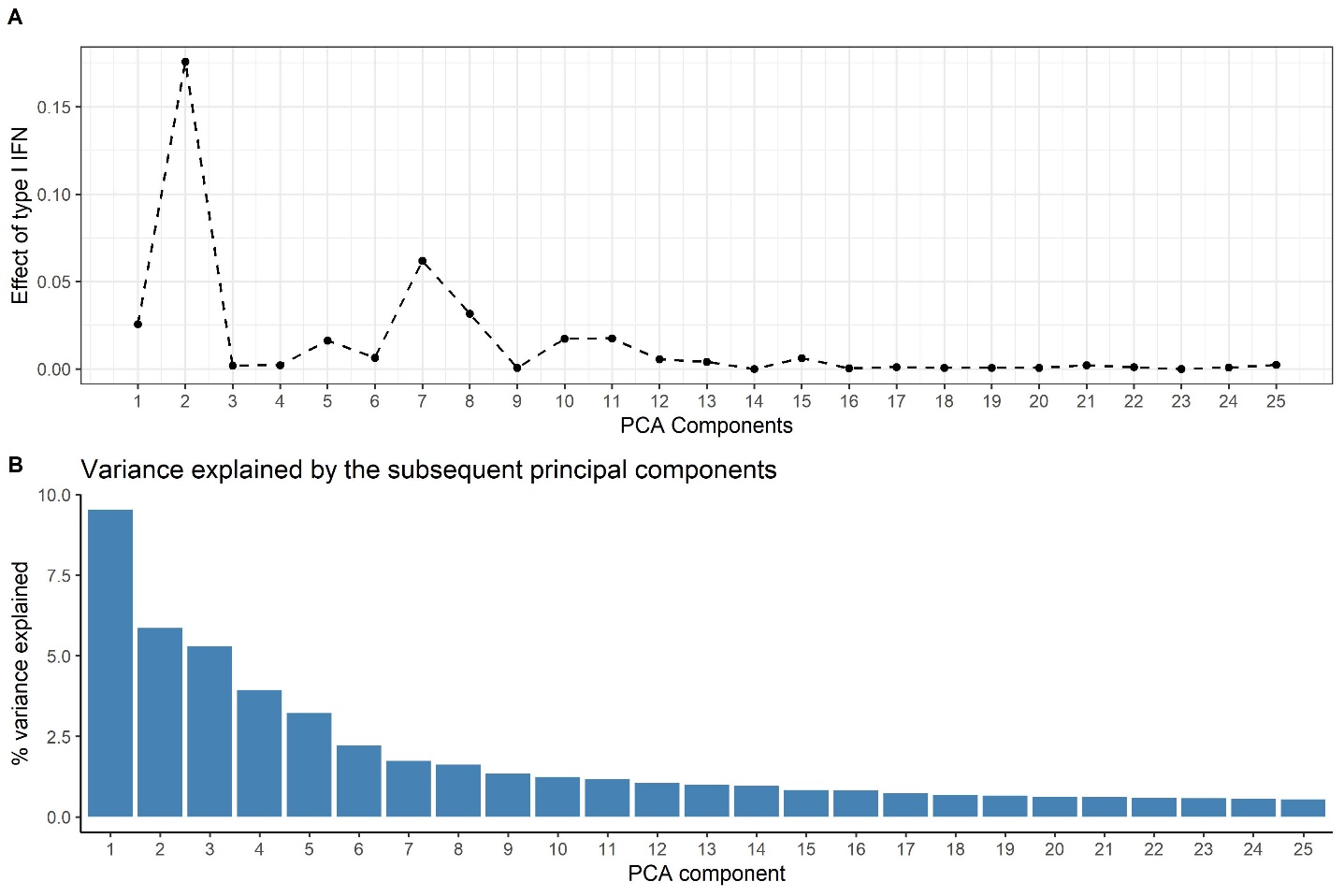


**Supplementary Figure 6: Variance explained by the subsequent principal components in PCA.** (A) Fraction of variance explained by IFN I status as a predictor for each of the first 25 PCs of the gene expression matrix from TB patients from the MDS calculated using 100-times randomization. PC2 and PC7 explain the biggest fraction of the variance. (B) Fraction of variance explained by the first 25 PCs of the gene expression matrix from TB patients from the MDS.


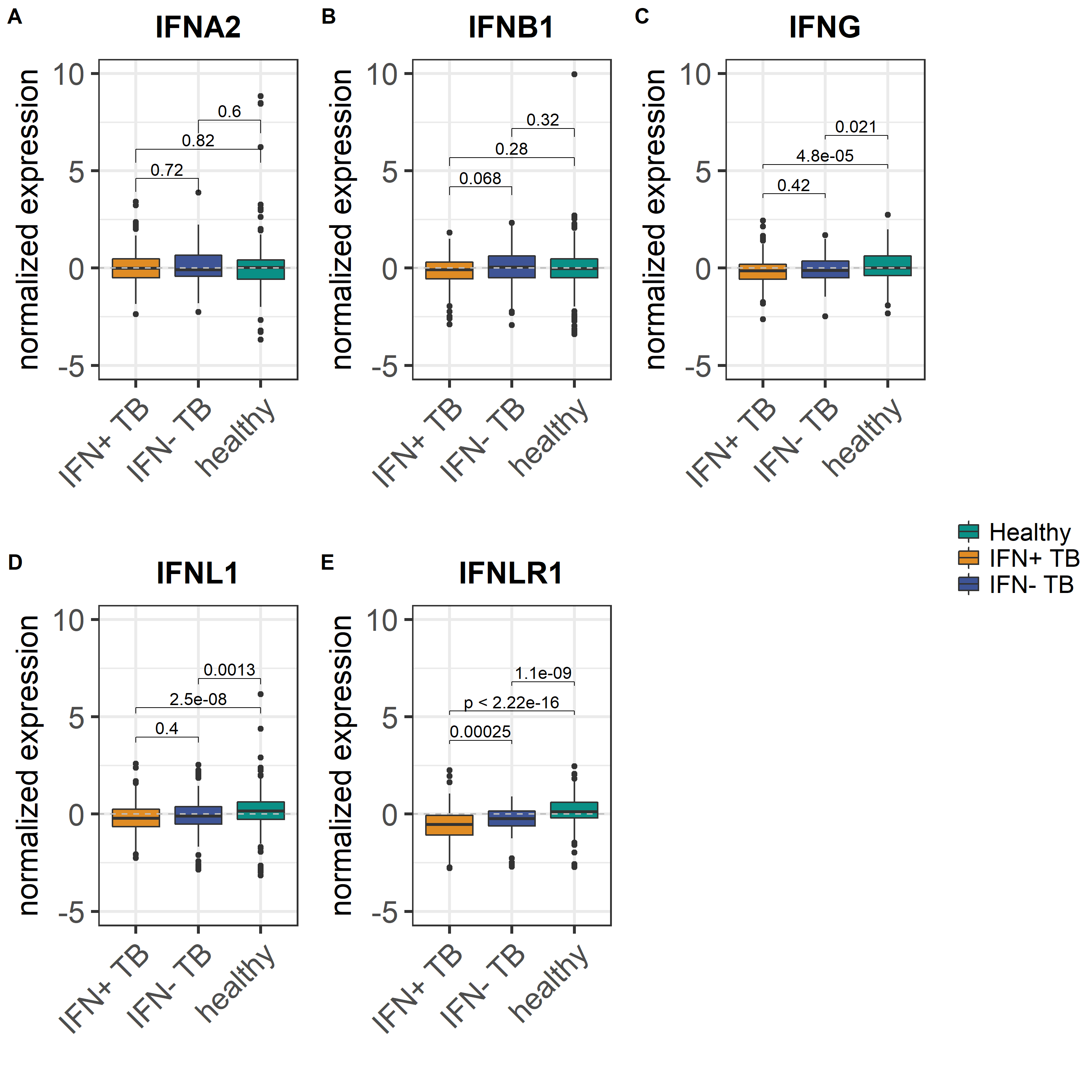


**Supplementary Figure 7: Expression of IFN type I, II and III genes: IFNA2 (A), IFNB1 (B), IFNG (C), IFNL1 (D) and IFN λ receptor gene IFNLR1 (E).**


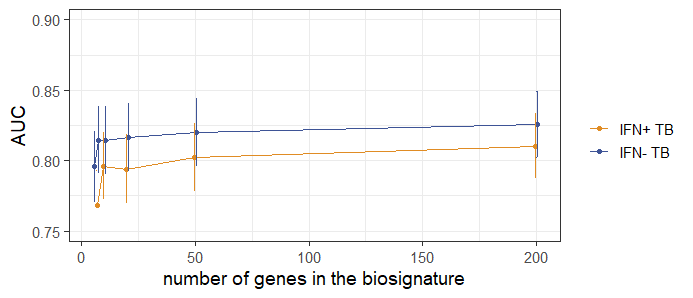


**Supplementary Figure 8: Dependence of the AUC of RF TB patients classification on the number of genes in the signature.** The bars indicate 95% confidence intervals of calculated AUC. Improvement of signature’s AUC was chosen with signature size of 20 transcripts for IFN+ and 50 transcripts for IFN- signature.

 
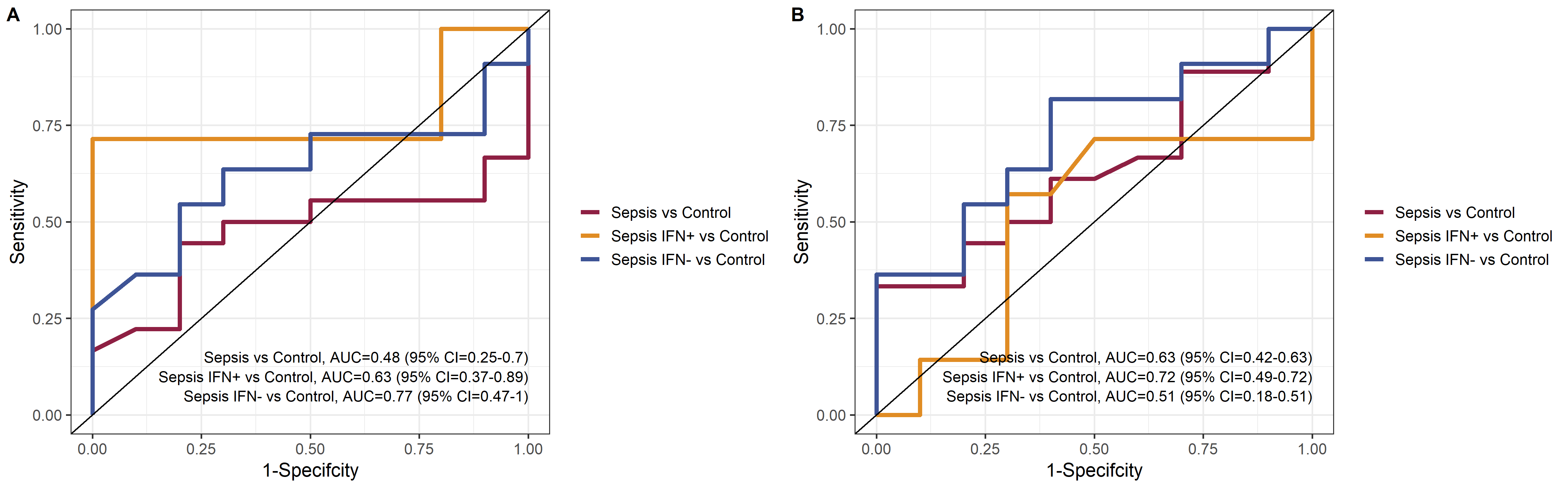


**Supplementary Figure 9: Performance of the TB signatures on the sepsis test MDS**. 20 transcript IFN+ TB signature (A) and 50 transcript IFN- TB signature (B) was tested on the sepsis test set. The TB IFN+ and IFN- signatures are not sensitive and specific towards detection of sepsis.


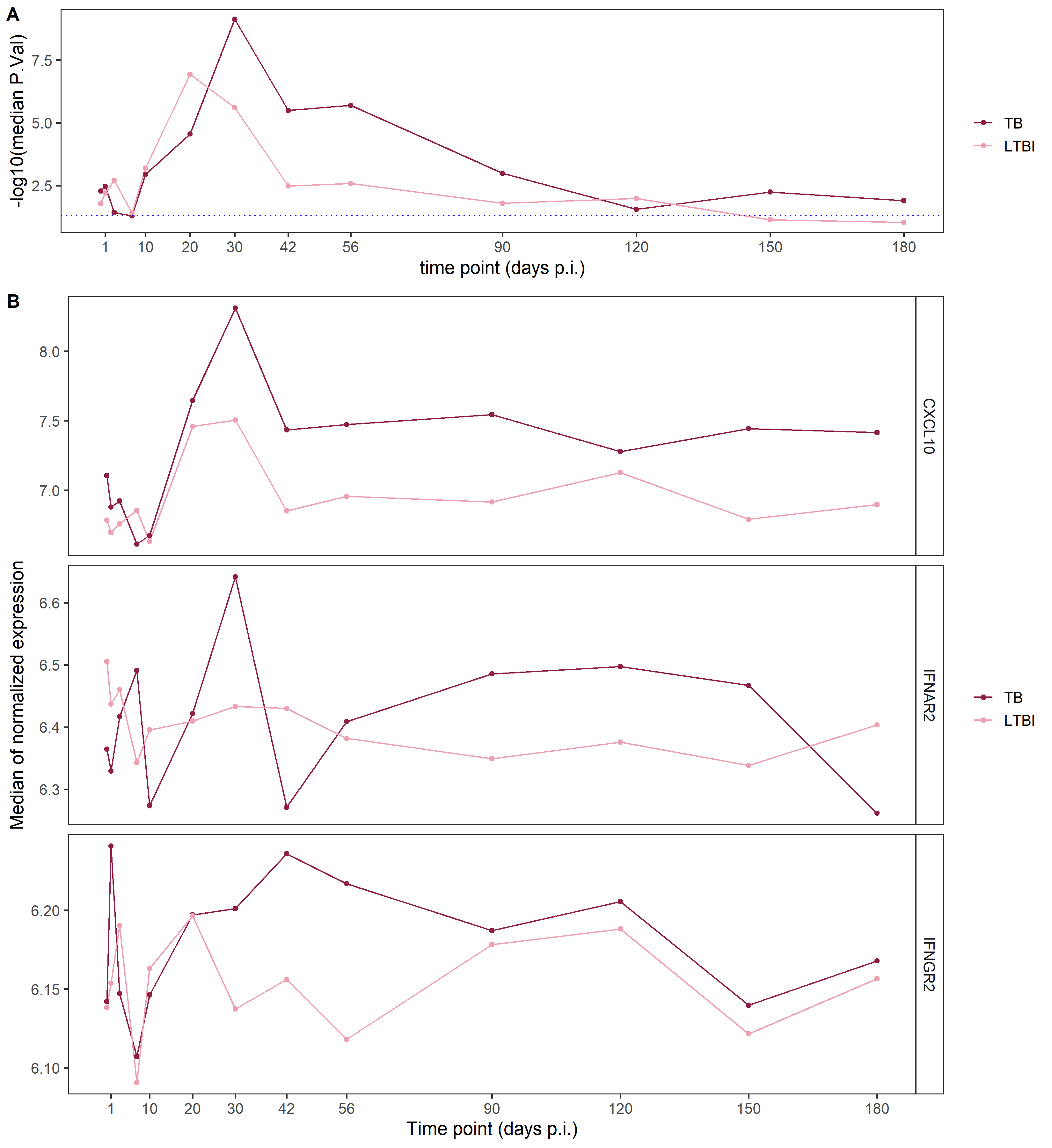


**Supplementary Figure 10:High level of IFN module enrichment tends to be prolonged in the animals with active TB.** Both the negative logarithm of median p-value for enrichment using CERNO test and IFN modules (A) as well as the median normalized expression of IFN inducible genes CXCL10, IFNAR2 and IFNGR2 (B) present the trend of higher peak in the animals with active TB compared to LTBI as well as prolonged elevated value after the peak in the days 20-42.

## Supplementary Tables:

**Supplementary Table 1: Signature transcripts of IFN+ and IFN- TB patients.** The transcript ENSEMBL IDs and corresponding HGNC gene names are given. The third column indicates the transcripts overlapping in the IFN+ and IFN- biosignature. The last column indicates transcripts which were indicated as IFN type I inducible or inducible by both IFN type I and type II by Interferome database.

| ENSEMBL ID | HGNC symbol | IFN-/IFN+ TB Signature overlap | Present in IFN I or IFN I and II module |
| --- | --- | --- | --- |
| ENSG00000152766 | ANKRD22 |  | + |
| ENSG00000154451 | GBP5 | + | + |
| ENSG00000163568 | AIM2 | + | + |
| ENSG00000198019 | FCGR1B |  | + |
| ENSG00000120217 | CD274 |  | + |
| ENSG00000168062 | BATF2 |  | + |
| ENSG00000115415 | STAT1 |  | + |
| ENSG00000162645 | GBP2 | + | + |
| ENSG00000070501 | POLB | + | + |
| ENSG00000140105 | WARS1 | + |  |
| ENSG00000145685 | LHFPL2 | + |  |
| ENSG00000221963 | APOL6 |  | + |
| ENSG00000185338 | SOCS1 |  | + |
| ENSG00000108387 | SEPTIN4 |  |  |
| ENSG00000227816 | TAP1 |  | + |
| ENSG00000276336 | SCARF1 |  |  |
| ENSG00000099860 | GADD45B |  |  |
| ENSG00000108861 | DUSP3 | + |  |
| ENSG00000128203 | ASPHD2 |  | + |
| ENSG00000197646 | PDCD1LG2 |  |  |
| ENSG00000174944 | P2RY14 |  | + |
| ENSG00000068831 | RASGRP2 |  |  |
| ENSG00000142327 | RNPEPL1 |  |  |
| ENSG00000154451 | GBP5 | + | + |
| ENSG00000163568 | AIM2 | + | + |
| ENSG00000197024 | ZNF398 |  |  |
| ENSG00000066923 | STAG3 |  |  |
| ENSG00000103653 | CSK | + |  |
| ENSG00000136573 | BLK |  |  |
| ENSG00000111331 | OAS3 |  | + |
| ENSG00000162645 | GBP2 | + | + |
| ENSG00000288185 | IL27RA |  | + |
| ENSG00000258555 | SPECC1L-ADORA2A |  | + |
| ENSG00000184979 | USP18 |  | + |
| ENSG00000055332 | EIF2AK2 |  | + |
| ENSG00000070501 | POLB | + | + |
| ENSG00000127528 | KLF2 |  |  |
| ENSG00000288213 | MCRIP1 |  |  |
| ENSG00000181788 | SIAH2 |  |  |
| ENSG00000140105 | WARS1 | + |  |
| ENSG00000187231 | SESTD1 |  |  |
| ENSG00000185112 | FAM43A |  |  |
| ENSG00000276045 | ORAI1 |  |  |
| ENSG00000153827 | TRIP12 |  |  |
| ENSG00000167371 | PRRT2 |  |  |
| ENSG00000145685 | LHFPL2 |  |  |
| ENSG00000158669 | GPAT4 |  |  |
| ENSG00000119471 | HSDL2 |  |  |
| ENSG00000132185 | FCRLA |  |  |
| ENSG00000186625 | KATNA1 |  |  |
| ENSG00000111837 | MAK |  | + |
| ENSG00000138363 | ATIC |  |  |
| ENSG00000114541 | FRMD4B |  | + |
| ENSG00000103740 | ACSBG1 |  |  |
| ENSG00000119457 | SLC46A2 |  |  |
| ENSG00000091490 | SEL1L3 |  | + |
| ENSG00000101079 | NDRG3 |  |  |
| ENSG00000136436 | CALCOCO2 |  | + |
| ENSG00000284889 | PSME2 |  | + |
| ENSG00000119655 | NPC2 |  | + |
| ENSG00000112624 | BICRAL |  |  |
| ENSG00000159248 | GJD2 |  |  |
| ENSG00000242361 | HLA-DMA |  | + |
| ENSG00000162434 | JAK1 |  |  |
| ENSG00000108861 | DUSP3 | + |  |
| ENSG00000168003 | SLC3A2 |  |  |
| ENSG00000135074 | ADAM19 |  |  |
| ENSG00000189159 | JPT1 |  |  |
| ENSG00000198130 | HIBCH |  |  |
| ENSG00000144645 | OSBPL10 |  |  |

**Supplementary Table 2: List of all gene sets used in the study sorted by the r value of correlation of eigengenes of PC1 of a given gene set with eigengene of PC1 of IFN gene set**. The p-value from chi^2^ test for proportion of enrichment of a given module and enrichment of IFN gene set in TB patients is also given.

| Gene set ID | Gene set name | p-value (chi^2^ test) | r (eigengene correlation) | collection |
| --- | --- | --- | --- | --- |
| M5950 | Hallmark allograft rejection | 1.4E-20 | 0.98 | HALLMARK |
| M5939 | Hallmark p53 pathway | 1.5E-17 | 0.98 | HALLMARK |
| hsa04064 | NF-kappa B signaling pathway | 2.5E-10 | 0.96 | KEGG |
| hsa04210 | Apoptosis | 3.4E-06 | 0.96 | KEGG |
| M5947 | Hallmark il2 stat5 signaling | 9.5E-20 | 0.96 | HALLMARK |
| LI.M5.0 | regulation of antigen presentation and immune response | 1.2E-18 | 0.96 | TMOD |
| M5921 | Hallmark complement | 2.1E-20 | 0.96 | HALLMARK |
| hsa05235 | PD-L1 expression and PD-1 checkpoint pathway in cancer | 2.4E-16 | 0.96 | KEGG |
| M5953 | Hallmark kras signaling up | 3.0E-08 | 0.96 | HALLMARK |
| hsa05166 | Human T-cell leukemia virus 1 infection | 3.4E-11 | -0.96 | KEGG |
| LI.M37.0 | immune activation - generic cluster | 1.3E-15 | 0.95 | TMOD |
| hsa05203 | Viral carcinogenesis | 2.3E-07 | 0.95 | KEGG |
| M5902 | Hallmark apoptosis | 1.9E-17 | 0.95 | HALLMARK |
| M5932 | Hallmark inflammatory response | 2.8E-19 | 0.95 | HALLMARK |
| hsa05321 | Inflammatory bowel disease | 4.0E-07 | -0.95 | KEGG |
| hsa04659 | Th17 cell differentiation | 1.4E-09 | -0.94 | KEGG |
| hsa04660 | T cell receptor signaling pathway | 7.4E-10 | -0.94 | KEGG |
| hsa04217 | Necroptosis | 9.5E-07 | 0.93 | KEGG |
| LI.M16 | TLR and inflammatory signaling | 3.3E-14 | 0.93 | TMOD |
| DC.M6.13 | Cell Death | 1.2E-19 | 0.93 | TMOD |
| hsa04625 | C-type lectin receptor signaling pathway | 3.7E-08 | 0.93 | KEGG |
| hsa04658 | Th1 and Th2 cell differentiation | 1.6E-09 | -0.93 | KEGG |
| hsa05169 | Epstein-Barr virus infection | 1.8E-13 | 0.93 | KEGG |
| hsa05162 | Measles | 2.2E-07 | 0.93 | KEGG |
| hsa04640 | Hematopoietic cell lineage | 1.3E-05 | -0.93 | KEGG |
| LI.M3 | regulation of signal transduction | 2.0E-13 | 0.93 | TMOD |
| DC.M3.2 | Inflammation | 1.8E-10 | 0.92 | TMOD |
| LI.S5 | DC surface signature | 1.8E-05 | 0.92 | TMOD |
| M5890 | Hallmark tnfa signaling via nfkb | 2.5E-22 | 0.92 | HALLMARK |
| hsa05132 | Salmonella infection | 1.6E-08 | 0.92 | KEGG |
| hsa05131 | Shigellosis | 1.2E-07 | 0.92 | KEGG |
| M5938 | Hallmark reactive oxygen species pathway | 1.4E-07 | 0.92 | HALLMARK |
| M5897 | Hallmark il6 jak stat3 signaling | 4.2E-13 | 0.92 | HALLMARK |
| hsa04650 | Natural killer cell mediated cytotoxicity | 6.7E-05 | 0.91 | KEGG |
| hsa05145 | Toxoplasmosis | 4.0E-07 | 0.91 | KEGG |
| hsa04662 | B cell receptor signaling pathway | 2.0E-05 | 0.91 | KEGG |
| LI.M118.0 | enriched in monocytes (IV) | 1.6E-13 | 0.91 | TMOD |
| LI.M84 | integrins and cell adhesion | 4.5E-06 | 0.91 | TMOD |
| LI.M37.1 | enriched in neutrophils (I) | 5.6E-08 | 0.91 | TMOD |
| DC.M4.6 | Inflammation | 1.3E-17 | 0.91 | TMOD |
| hsa05152 | Tuberculosis | 7.6E-07 | 0.91 | KEGG |
| LI.M109 | receptors, cell migration | 1.8E-09 | 0.91 | TMOD |
| LI.M36 | T cell surface, activation | 3.6E-13 | 0.90 | TMOD |
| hsa04145 | Phagosome | 5.9E-05 | 0.90 | KEGG |
| DC.M4.2 | Inflammation | 1.6E-10 | 0.90 | TMOD |
| hsa05164 | Influenza A | 2.5E-11 | 0.90 | KEGG |
| LI.S4 | Monocyte surface signature | 2.0E-12 | 0.89 | TMOD |
| hsa04380 | Osteoclast differentiation | 2.2E-09 | 0.89 | KEGG |
| LI.S11 | Activated (LPS) dendritic cell surface signature | 3.3E-06 | 0.89 | TMOD |
| hsa05170 | Human immunodeficiency virus 1 infection | 2.6E-07 | 0.89 | KEGG |
| LI.M44 | T cell signaling and costimulation | 2.6E-12 | -0.89 | TMOD |
| LI.M11.0 | enriched in monocytes (II) | 4.9E-17 | 0.89 | TMOD |
| hsa05142 | Chagas disease | 5.5E-08 | 0.88 | KEGG |
| hsa05140 | Leishmaniasis | 1.3E-07 | 0.88 | KEGG |
| hsa04668 | TNF signaling pathway | 1.7E-07 | 0.88 | KEGG |
| hsa05133 | Pertussis | 1.8E-10 | 0.88 | KEGG |
| DC.M4.15 | T cells | 1.7E-09 | -0.88 | TMOD |
| DC.M4.13 | Inflammation | 5.4E-04 | 0.88 | TMOD |
| hsa04621 | NOD-like receptor signaling pathway | 2.8E-14 | 0.88 | KEGG |
| LI.M31 | cell cycle and growth arrest | 2.8E-09 | 0.88 | TMOD |
| LI.M53 | inflammasome receptors and signaling | 7.4E-08 | 0.87 | TMOD |
| LI.M64 | enriched in activated dendritic cells/monocytes | 3.3E-07 | 0.87 | TMOD |
| LI.S0 | T cell surface signature | 2.3E-09 | -0.87 | TMOD |
| LI.M11.1 | blood coagulation | 1.6E-08 | 0.87 | TMOD |
| LI.M43.1 | myeloid, dendritic cell activation via NFkB (II) | 3.3E-07 | 0.87 | TMOD |
| LI.M5.1 | T cell activation and signaling | 1.9E-09 | -0.87 | TMOD |
| LI.M117 | cell adhesion (GO) | 6.9E-16 | -0.86 | TMOD |
| LI.M43.0 | myeloid, dendritic cell activation via NFkB (I) | 4.6E-07 | 0.86 | TMOD |
| hsa04666 | Fc gamma R-mediated phagocytosis | 1.3E-07 | 0.86 | KEGG |
| hsa05221 | Acute myeloid leukemia | 1.6E-05 | 0.86 | KEGG |
| LI.M7.1 | T cell activation (I) | 1.1E-10 | -0.86 | TMOD |
| LI.M73 | enriched in monocytes (III) | 4.6E-07 | 0.86 | TMOD |
| LI.M7.0 | enriched in T cells (I) | 9.5E-10 | -0.86 | TMOD |
| hsa05160 | Hepatitis C | 1.9E-06 | 0.86 | KEGG |
| hsa05034 | Alcoholism | 2.4E-04 | 0.85 | KEGG |
| LI.M81 | enriched in myeloid cells and monocytes | 8.7E-05 | 0.85 | TMOD |
| LI.M223 | enriched in T cells (II) | 6.4E-08 | -0.85 | TMOD |
| hsa05135 | Yersinia infection | 2.5E-06 | 0.85 | KEGG |
| hsa05340 | Primary immunodeficiency | 2.2E-05 | -0.85 | KEGG |
| LI.M7.4 | T cell activation (III) | 2.6E-11 | -0.85 | TMOD |
| LI.M61.2 | enriched in NK cells (receptor activation) | 7.8E-08 | -0.85 | TMOD |
| LI.M18 | T cell differentiation via ITK and PKC | 1.0E-11 | -0.84 | TMOD |
| LI.M12 | CD28 costimulation | 1.1E-03 | -0.84 | TMOD |
| DC.M5.1 | Inflammation | 1.0E-18 | 0.84 | TMOD |
| hsa04620 | Toll-like receptor signaling pathway | 2.6E-07 | 0.84 | KEGG |
| LI.M168 | enriched in dendritic cells | 3.4E-07 | 0.84 | TMOD |
| M5935 | Hallmark fatty acid metabolism | 1.2E-04 | -0.83 | HALLMARK |
| DC.M4.7 | Cell Cycle | 3.8E-12 | -0.83 | TMOD |
| LI.M19 | T cell differentiation (Th2) | 8.3E-09 | -0.83 | TMOD |
| LI.M14 | T cell differentiation | 4.8E-12 | -0.82 | TMOD |
| LI.M40 | complement and other receptors in DCs | 6.6E-23 | 0.82 | TMOD |
| DC.M6.12 | Mitochondrial Stress | 1.3E-04 | -0.82 | TMOD |
| LI.M126 | double positive thymocytes | 7.3E-09 | -0.81 | TMOD |
| hsa05322 | Systemic lupus erythematosus | 8.1E-08 | 0.81 | KEGG |
| LI.M7.3 | T cell activation (II) | 1.8E-10 | -0.80 | TMOD |
| DC.M4.3 | Protein Synthesis | 1.9E-06 | -0.80 | TMOD |
| DC.M3.5 | Cell Cycle | 5.1E-04 | -0.80 | TMOD |
| LI.M245 | translation initiation factor 3 complex | 5.2E-08 | -0.80 | TMOD |
| M5924 | Hallmark mtorc1 signaling | 1.1E-06 | -0.79 | HALLMARK |
| M5926 | Hallmark myc targets v1 | 9.6E-12 | -0.79 | HALLMARK |
| hsa03008 | Ribosome biogenesis in eukaryotes | 8.0E-02 | -0.79 | KEGG |
| M5898 | Hallmark dna repair | 4.3E-03 | -0.79 | HALLMARK |
| LI.M4.0 | cell cycle and transcription | 1.6E-08 | -0.79 | TMOD |
| DC.M7.1 | Inflammation | 6.2E-12 | 0.78 | TMOD |
| LI.M45 | leukocyte activation and migration | 3.8E-05 | -0.78 | TMOD |
| LI.M61.0 | enriched in NK cells (II) | 3.6E-06 | -0.78 | TMOD |
| LI.M4.13 | cell junction (GO) | 1.8E-05 | 0.76 | TMOD |
| hsa05168 | Herpes simplex virus 1 infection | 7.4E-08 | -0.76 | KEGG |
| hsa03013 | RNA transport | 7.5E-04 | -0.76 | KEGG |
| DC.M6.6 | Apoptosis / Survival | 4.4E-11 | 0.76 | TMOD |
| LI.M13 | innate activation by cytosolic DNA sensing | 9.5E-11 | 0.76 | TMOD |
| DC.M4.1 | T cell | 2.2E-14 | -0.75 | TMOD |
| hsa04142 | Lysosome | 1.8E-06 | 0.75 | KEGG |
| M5922 | Hallmark unfolded protein response | 1.6E-08 | -0.75 | HALLMARK |
| hsa00970 | Aminoacyl-tRNA biosynthesis | 1.2E-02 | -0.75 | KEGG |
| LI.M65 | IL2, IL7, TCR network | 1.4E-07 | -0.75 | TMOD |
| DC.M5.7 | Inflammation | 3.0E-02 | 0.74 | TMOD |
| LI.M7.2 | enriched in NK cells (I) | 1.5E-04 | -0.73 | TMOD |
| M5928 | Hallmark myc targets v2 | 7.4E-04 | -0.73 | HALLMARK |
| LI.M157 | enriched in NK cells (III) | 6.3E-03 | -0.73 | TMOD |
| DC.M5.12 | Interferon | 9.5E-40 | 0.72 | TMOD |
| LI.M4.3 | myeloid cell enriched receptors and transporters | 2.9E-05 | 0.72 | TMOD |
| hsa05020 | Prion disease | 2.4E-01 | -0.70 | KEGG |
| M5936 | Hallmark oxidative phosphorylation | 1.1E-01 | -0.70 | HALLMARK |
| DC.M5.10 | Mitochondrial Respiration | 5.4E-01 | -0.69 | TMOD |
| hsa03010 | Ribosome | 7.4E-04 | -0.69 | KEGG |
| LI.M209 | lysosome | 6.9E-05 | 0.68 | TMOD |
| LI.M111.0 | viral sensing & immunity; IRF2 targets network (I) | 1.3E-14 | 0.68 | TMOD |
| M5913 | Hallmark interferon gamma response | 4.3E-43 | 0.67 | HALLMARK |
| DC.M5.9 | Protein Synthesis | 3.3E-06 | -0.67 | TMOD |
| LI.M112.0 | complement activation (I) | 4.2E-17 | 0.67 | TMOD |
| M5925 | Hallmark e2f targets | 3.1E-03 | -0.67 | HALLMARK |
| LI.M165 | enriched in activated dendritic cells (II) | 2.8E-14 | 0.66 | TMOD |
| DC.M3.6 | Cytotoxic/NK Cell | 1.8E-02 | -0.66 | TMOD |
| DC.M8.83 | Immune Responses | 1.8E-04 | -0.65 | TMOD |
| LI.M67 | activated dendritic cells | 4.4E-22 | 0.63 | TMOD |
| LI.M156.0 | plasma cells & B cells, immunoglobulins | 2.5E-02 | -0.62 | TMOD |
| LI.M47.2 | enriched in B cells (III) | 2.2E-01 | -0.62 | TMOD |
| DC.M3.4 | Interferon | 1.2E-30 | 0.61 | TMOD |
| M5901 | Hallmark g2m checkpoint | 1.5E-06 | -0.59 | HALLMARK |
| M5911 | Hallmark interferon alpha response | 3.3E-26 | 0.58 | HALLMARK |
| hsa05012 | Parkinson disease | 8.9E-01 | -0.58 | KEGG |
| hsa04612 | Antigen processing and presentation | 1.5E-04 | -0.58 | KEGG |
| LI.M111.1 | viral sensing & immunity; IRF2 targets network (II) | 1.5E-18 | 0.57 | TMOD |
| LI.M75 | antiviral IFN signature | 2.9E-23 | 0.57 | TMOD |
| DC.M4.10 | B cell | 6.2E-03 | -0.57 | TMOD |
| LI.M68 | RIG-1 like receptor signaling | 5.9E-08 | 0.55 | TMOD |
| LI.M69 | enriched in B cells (VI) | 5.7E-01 | -0.54 | TMOD |
| LI.M47.1 | enriched in B cells (II) | 6.8E-02 | -0.53 | TMOD |
| LI.M127 | type I interferon response | 3.3E-20 | 0.53 | TMOD |
| LI.M47.0 | enriched in B cells (I) | 3.4E-01 | -0.53 | TMOD |
| LI.M199 | platelet activation & blood coagulation | 6.4E-01 | 0.51 | TMOD |
| LI.M150 | innate antiviral response | 1.8E-12 | 0.51 | TMOD |
| DC.M5.6 | Mitochondrial Stress / Proteasome | 5.1E-01 | -0.48 | TMOD |
| LI.M196 | platelet activation - actin binding | 9.2E-01 | 0.48 | TMOD |
| DC.M1.1 | Platelets | 4.2E-01 | 0.47 | TMOD |
| DC.M5.15 | Neutrophils | 1.4E-06 | 0.44 | TMOD |
| DC.M1.2 | Interferon | 5.6E-16 | 0.44 | TMOD |
| LI.M4.1 | cell cycle (I) | 4.1E-01 | -0.36 | TMOD |
| LI.M124 | enriched in membrane proteins | 7.9E-03 | 0.35 | TMOD |
| LI.M4.5 | mitotic cell cycle in stimulated CD4 T cells | 1.4E-01 | -0.33 | TMOD |
| LI.M4.2 | PLK1 signaling events | 7.7E-01 | -0.29 | TMOD |
| DC.M3.3 | Cell Cycle | 1.0E-01 | -0.22 | TMOD |
| DC.M4.14 | Monocytes | 1.1E-01 | 0.17 | TMOD |
| M5945 | Hallmark heme metabolism | 2.0E-01 | 0.13 | HALLMARK |
| DC.M2.3 | Erythrocytes | 3.9E-01 | 0.12 | TMOD |
| DC.M3.1 | Erythrocytes | 9.6E-01 | 0.08 | TMOD |
| DC.M6.18 | Erythrocytes | 3.9E-01 | 0.03 | TMOD |
